# Bypassing Endocytic Barriers: Visualizing Membrane Fusion and Endosomal Escape of Cubic Phase Lipid Nanoparticles (Cubosomes)

**DOI:** 10.1002/smll.202502231

**Published:** 2025-07-25

**Authors:** Sue Lyn Yap, Chaitali Dekiwadia, Enrico Della Gaspera, Calum J. Drummond, Charlotte E. Conn, Nhiem Tran

**Affiliations:** ^1^ School of Science STEM College RMIT University 124 La Trobe St Melbourne VIC 3000 Australia; ^2^ RMIT Microscopy & Microanalysis Facility (RMMF) STEM College RMIT University 124 La Trobe St Melbourne VIC 3000 Australia

**Keywords:** cubosomes, endosomal escape, lipid nanoparticles, membrane fusion, microscopy, nanoparticle–cell interactions

## Abstract

Lipid nanoparticles (LNPs) are versatile platforms for drug delivery, offering solutions for targeted therapeutics, gene therapy, and vaccines. Cubosomes ‐ cubic phase lipid nanoparticles ‐ exhibit unique structural properties that may allow direct cytosolic delivery, bypassing endocytic pathways that often limit intracellular drug delivery. Despite their potential, the mechanisms of cubosome interactions with mammalian cells, particularly their membrane fusion behavior, remain unclear. This study employs a multimodal microscopy‐based approach to investigate cubosome interactions with cells, focusing on membrane fusion, lipid exchange, and endosomal escape. Advanced imaging techniques, including transmission, scanning, and cryogenic scanning electron microscopy, and live‐cell fluorescence imaging, are used alongside tailored cubosome variants to examine interactions at micro‐ and nanoscale dimensions. Cubosome behaviors are compared to other nanoparticleslike liposomes and gold nanoparticles. For the first time, membrane fusion with mammalian plasma and endosomal membranes is visualized at the nanoscale, revealing how cubosomes bypass conventional endocytic pathways to deliver cargo directly into the cytosol. This work provides critical insights into LNP–cell interactions, establishing cubosomes as promising candidates for overcoming endosomal escape limitations in drug delivery. These findings will aid in developing next‐generation lipid‐based nanocarriers, particularly for RNA therapeutics, where efficient cytosolic delivery is essential for therapeutic efficacy.

## Graphical abstract

This study uses advanced microscopy techniques, including transmission electron microscopy, scanning electron microscopy (SEM), cryogenic SEM, and live‐cell fluorescence imaging, to investigate lipid cubosome interactions with mammalian cells. It provides the first direct nanoscale evidence of cubosome membrane fusion with plasma and endosomal membranes, demonstrating their ability to bypass endocytic recycling and lysosomal degradation through lipid exchange and membrane disruption.

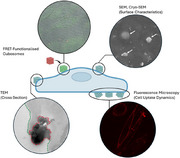

## 1 Introduction

In December 2020, the Food and Drug Administration granted emergency use authorization for two lipid nanoparticle (LNP)‐based vaccine formulations designed to deliver mRNA encoding the SARS‐COV‐2 spike protein, highlighting the transformative potential of LNPs in medicine. While the implementation of LNP technology in mRNA vaccines marks a significant scientific milestone, lipid‐based RNA delivery systems are relatively novel in bioactive agent delivery.^[^ ^1^ ^]^

LNPs are derived from lipid mesophases, which self‐assemble in aqueous environments through the hydrophobic effect, driven by their amphiphilic nature.^[^ ^2^ ^]^ The specific molecular architecture of the lipid components further dictates the formation of diverse 1D, 2D, and 3D nanostructures, such as the lamellar phase, inverse bicontinuous cubic phases, and the inverse hexagonal phase,^[^ ^2^ ^]^ which play pivotal roles in encapsulating and delivering therapeutic cargo. While liposomal formulations dominate clinically approved lipid‐based nanomedicines,^[^ ^3^ ^]^ there is a recent trend toward more complex nanoformulations for improved drug delivery.

Nanoparticles are often internalized into cells via endocytosis, where they are wholly internalized and enclosed within endosomal vesicles.^[^ ^4^ ^]^ Therefore, the effectiveness of LNP delivery remains constrained by endocytic recycling and relies on the successful escape of LNPs from endosomes into the cytosol. Studies have reported that less than 5% of nucleic acids or siRNA escape endocytic recycling post‐LNP endocytosis,^[^ ^5^, ^6^ ^]^ highlighting a critical need for new strategies that improve intracellular delivery efficiency.

LNPs possessing an inverse bicontinuous cubic internal nanostructure, termed “cubosomes,” offer an alternative intracellular delivery mechanism through membrane fusion with cellular membranes.^[^ ^7^, ^8^, ^9^ ^]^ Unlike endocytosis, membrane fusion enables direct lipid mixing between LNPs and cell membranes, facilitating cargo delivery into the cytosol while bypassing endosomal entrapment.^[^ ^7^, ^10^ ^]^ Cubosomes have been extensively studied as nanocarriers due to their advantageous properties, including enhanced encapsulation efficiency^[^ ^11^, ^12^ ^]^ and delivery of bioactive cargo,^[^ ^13^, ^14^, ^15^, ^16^ ^]^ along with their biocompatibility and biodegradability.^[^ ^12^, ^17^, ^18^ ^]^

Dyett et al. previously investigated the fusion dynamics of cubosomes with model cell membranes and observed similar behaviors in small intestine and STO cell lines, suggesting that cubosomes interact with cells primarily via membrane fusion.^[^ ^7^ ^]^ In our previous study, we demonstrated that cubosomes were predominantly internalized through passive nonendocytic pathways—likely membrane fusion—which we believe contributed to their superior cellular uptake compared to liposomes, hexosomes, and micellar cubosomes of similar size, surface charge, and composition.^[^ ^19^ ^]^ Collectively, these studies and others highlight the superior fusogenic activity of cubosomes, however, direct visualization of cubosome membrane fusion with mammalian cells has remained elusive.

The nanometer scale at which cubosomes exist poses significant technical challenges for directly visualizing their interactions with cells. Previous studies on cubosome–cell interactions have primarily relied on light and fluorescence microscopy methods, with a heavier emphasis on comparative models such as model membranes to indirectly compare cell membrane dynamics.^[^ ^7^, ^20^, ^21^ ^]^ However, these methods are unable to directly visualize interactions between LNPs and mammalian cellular membranes, leaving a critical gap in direct evidence of cubosome membrane fusion. Conventional electron microscopy methods provide a viable solution and have historically been widely used to observe mammalian cell interactions due to their ease in cell sample processing, enabling nanoscale visualization.^[^ ^22^ ^]^

In this study, we investigate cubosome–cell interactions using a suite of microscopy techniques, including confocal fluorescence microscopy, scanning electron microscopy (SEM), transmission electron microscopy (TEM), and cryogenic scanning electron microscopy (Cryo‐SEM). Additionally, varying cubosome formulations were optimized to uncover distinct mechanistic information into their cellular interactions. This multimodal approach integrates complementary methodologies to provide an unprecedented level of detail on the in vitro behaviors of cubosomes, capturing both micro‐ and nanoscale interactions.

At the micrometer scale, confocal microscopy is used to study the dynamic interactions of fluorescently labeled cubosomes and liposomes with cells, offering comparative insights into their differing uptake mechanisms. Additionally, we introduce a dual‐fluorescence‐labeled cubosome formulation that enables real‐time tracking of membrane fusion through Förster resonance energy transfer (FRET)‐based fluorescence changes. At the nanometer scale, TEM and SEM provide direct visualization of cubosome cellular interactions, offering evidence of their fusogenic behavior with both plasma and endosomal membranes. Cryo‐SEM further supports these findings by imaging cubosome‐treated cell surfaces in their frozen hydrated state, preserving native structures while minimizing artifacts associated with conventional methods involving chemical fixation and dehydration. By leveraging advanced microscopy methods, this study bridges a key gap in our understanding of LNP–cell interactions, offering direct visualization of nonendocytic internalization mechanisms.

Endosomal escape remains one of the greatest challenges in LNP‐based therapeutics, as most nanoparticles are confined within endosomes and degraded before reaching the cytosol. Our findings suggest that cubosomes not only bypass extensive endosomal trafficking via membrane fusion but also facilitate lipid‐mediated endosomal destabilization, potentially promoting escape. This unique capability distinguishes cubosomes as a promising platform for enhancing intracellular drug delivery efficiency, particularly for RNA therapeutics, where efficient cytosolic delivery is paramount for effective gene expression.

## 2 Results and Discussion

### 2.1 Confocal Microscopy Reveals Distinct Cellular Interactions with Liposomes and Cubosomes

**Figure 1:**
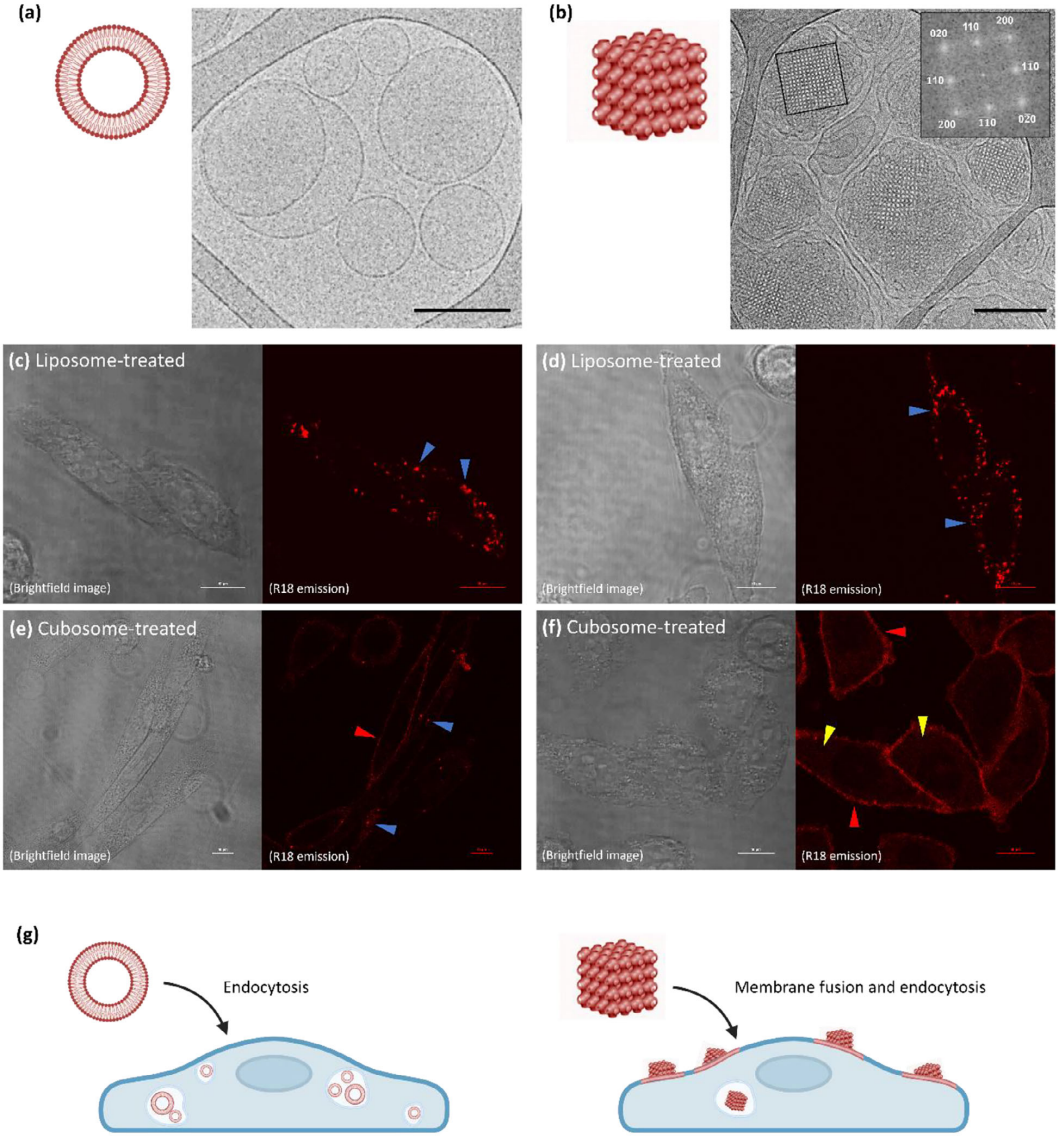
a) Cryo‐TEM of liposomes with an illustration of the liposome nanostructure, and b) cryo‐TEM of cubosomes with FFT analysis (inset) of the area within the black square showing characteristic *Im3m* reflections, and an illustration of cubosome nanostructure. Black scale bar = 200 nm. c,d) Confocal images illustrating the dynamics of fluorescently labeled liposomes and e,f) cubosomes. Bright punctate fluorescence (blue arrows) is observed in both liposome and cubosome‐treated cells. Membrane‐localized fluorescence (red arrows) and diffuse distributed fluorescence (yellow arrows) are observed in cubosome‐treated cells. Scale bars = 10 µm. g) Schematic illustration of the proposed interactions in liposome and cubosome‐treated cells, where liposomes are primarily endocytosed (bright punctate fluorescence) and cubosomes are internalized via both membrane fusion (membrane‐localized fluorescence) and endocytosis (bright punctate fluorescence).

To compare the cellular interactions and uptake dynamics visually, Chinese Hamster Ovarian (CHO) cells were incubated with fluorescently labeled liposomes and cubosomes for 1 h and imaged using confocal microscopy (**Figure** **1**). Both liposomes and cubosomes were labeled with 0.1% Octadecyl Rhodamine B Chloride (R18) and optimized to have similar size, surface charge, and surface stabilizer, while differing in their internal nanostructure. The average hydrodynamic diameters of both nanoparticles ranged between 200 and 250 nm and were essentially neutrally charged (Figures S1c–e and S2 (see accompanying text for Figure S2), Supporting Information). Small angle X‐ray scattering (SAXS) analysis and cryo‐TEM confirmed that cubosomes exhibited a primitive (*Im3m*) cubic phase, confirmed by characteristic Bragg peaks reflecting the √2, √4, √6 indices of the cubic *Im3m* space group^[^ ^17^ ^]^ in SAXS plots, and the characteristic *Im3m* reflections in the fast Fourier transform (FFT) analysis of cryo‐TEM images (Figure S1a,b, Supporting Information). Detailed information on the formulation and characterization of the R18‐labeled liposomes and cubosomes are provided in the Methods of the Supporting Information. For consistency, and due to their low polydispersity index (PDI) of 0.05 (Figure S1d, Supporting Information), R18‐cubosomes were used for all subsequent cell experiments that did not require additional cubosome functionalization, such as FRET‐based cubosomes or cubosomes loaded with <10 nm gold nanoparticles (Au_10_NPs).

In our previous study, liposomes were found to be primarily internalized via endocytosis, whereas cubosomes utilized multiple internalization pathways, including endocytosis (predominantly macropinocytosis) and a passive nonendocytic mechanism, likely membrane fusion.^[^ ^19^ ^]^ Consistent with these findings, confocal imaging revealed distinct uptake behaviors for liposome‐ and cubosome‐treated cells (Figure 1c–f). For liposome‐treated cells, fluorescence appeared as bright punctate spots confined within vesicles of varying sizes throughout the cytosol (Figure 1c,d). The heterogeneity in fluorescent spot sizes likely corresponds to endosomal vesicles of different pathways; where larger fluorescent spots may represent nanoparticle‐filled macropinosomes (which can range in size from 200 to 5000 nm^[^ ^23^, ^24^ ^]^), while smaller spots may correspond to endosomal vesicles with a lower size limit such as clathrin‐mediated endocytosis.

In contrast, cubosome‐treated cells exhibited heterogeneous fluorescence patterns, including plasma membrane‐localized fluorescence, bright punctate fluorescence (similar to liposomes), and diffuse cytosolic fluorescence (Figure 1e,f). These patterns align with the diverse internalization mechanisms employed by cubosomes, including both active endocytic pathways and passive nonendocytic pathways (membrane fusion). The membrane‐localized fluorescence observed (Figure 1e,f) is likely indicative of fluorescently labeled cubosomes undergoing membrane fusion (Figure 1g), while the bright punctate fluorescence observed in both liposome‐ and cubosome‐treated cells represents nanoparticle‐filled endosomes or lysosomes (Figure 1c–g). Notably, due to the resolution limit of confocal microscopy, we cannot be certain if the diffuse fluorescence is a result of endosomal escape and cubosome lipids in the cytosol, or the diffuse fluorescence originates from the cubosome–membrane fusion with the plasma membrane at the top of the cell. Collectively, these observations further validate the heterogeneous internalization mechanisms of cubosomes and highlight their unique fusogenic properties compared to liposomes^[^ ^9^, ^25^ ^]^ (Figure 1g).

### 2.2 FRET Reveals Membrane Fusion as a Key Interaction in Cubosome–Cell Dynamics

To investigate the membrane fusion of cubosomes with CHO cell membranes, the FRET pair Lissamine Rhodamine PE (L‐Rhod‐PE) and NBD‐PE were incorporated into cubosomes. SAXS analysis revealed that the FRET‐cubosomes showed a mixture of cubic *Im3m* (√2, √4, √6) and cubic *Pn3m* (√2, √3, √6, √8) cubic phases in water and cell media conditions (Figure S1b, Supporting Information). Their nanostructure was further validated by cryo‐TEM images and their FFT analysis (Figures S1a and S3b, Supporting Information). The FRET‐cubosomes had an average hydrodynamic diameter of 221 nm and were essentially neutrally charged (Figure S1c–e, Supporting Information). Cytotoxicity results can be found in the Supporting Information (Figure S4, Supporting Information).

**Figure 2:**
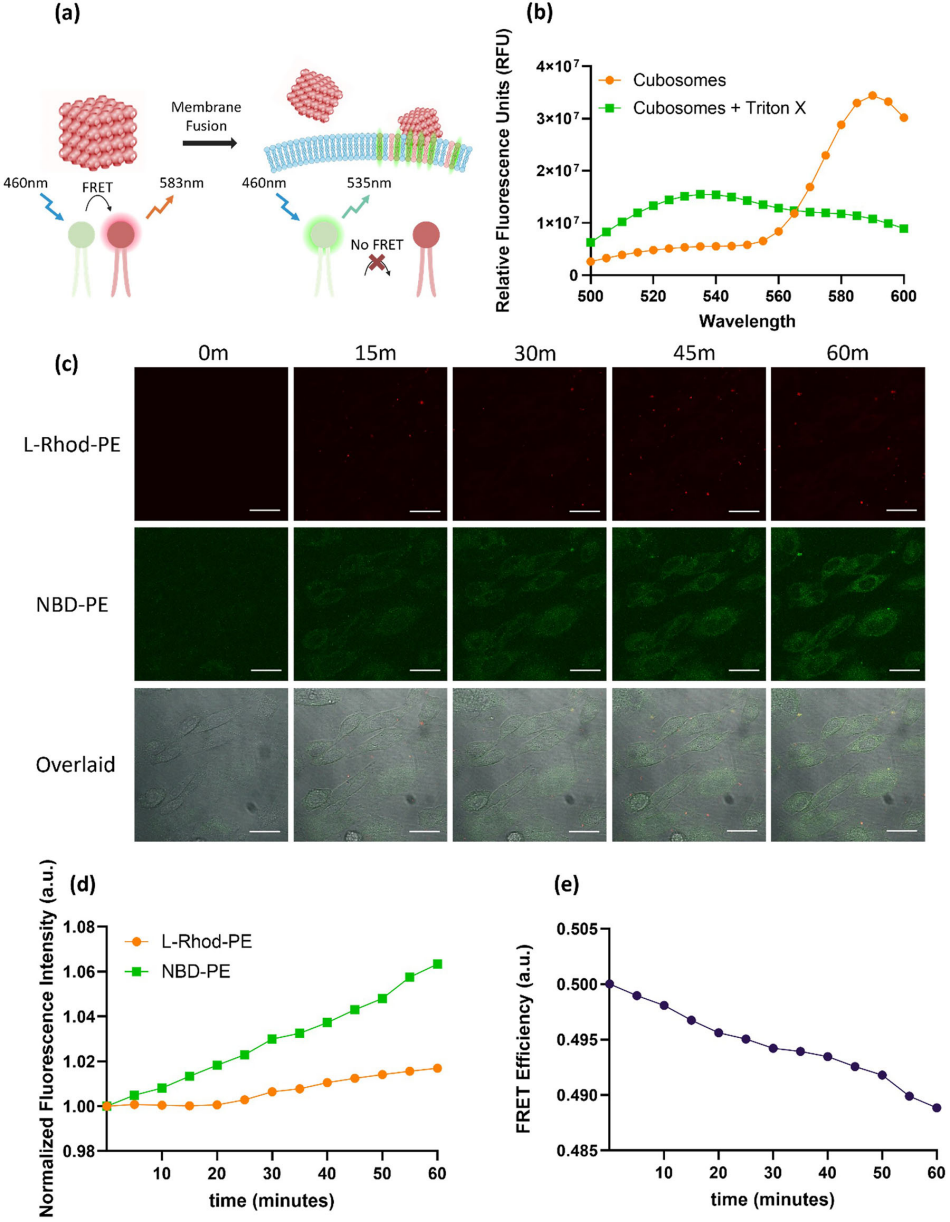
a) Illustration of FRET‐cubosome mechanism for intact cubosomes versus “fusing” cubosomes. b) Emission spectra of intact cubosomes and cubosomes solubilized with Triton‐X (a detergent), with an excitation wavelength of 460 nm (NBD‐PE excitation only). c) Confocal images of cubosome‐treated cells across 1 h of live cell imaging with L‐Rhod‐PE filter, NBD‐PE filter, and the overlaid images. *n* > 10. Scale bar = 25 µm. d) Normalized fluorescence intensity plots of L‐Rhod‐PE and NBD‐PE across 1 h of FRET‐cubosome treatment. An intensity threshold was applied in ImageJ to exclude bright punctate signals from free‐floating cubosomes, allowing quantification to focus on intracellular and membrane‐associated fluorescence within cells. e) FRET efficiency plot of cubosome‐treated cells across 1 h of treatment, where the FRET efficiency was calculated by dividing the L‐Rhod‐PE normalized fluorescence intensity by the total fluorescence intensity (L‐Rhod‐PE + NBD‐PE).

L‐Rhod‐PE has excitation and emission wavelengths of 560 and 583 nm, respectively, while NBD‐PE has excitation and emission wavelengths of 460 and 535 nm. The overlapping emission spectrum of NBD‐PE and excitation spectrum of L‐Rhod‐PE enables FRET when the two fluorophores are in close proximity (between 1 and 10 nm).^[^ ^26^, ^27^ ^]^ During FRET experiments, only the NBD‐PE excitation laser was used, and emissions from both fluorophores were collected and imaged. In intact cubosomes, where the FRET pair is closely associated, energy transfer occurs from the excited NBD‐PE to L‐Rhod‐PE, resulting in predominant emission from L‐Rhod‐PE (**Figure** **2**a). However, when the distance between the FRET pair increases, such as during cubosome–cell membrane fusion, FRET decreases and only NBD‐PE emits fluorescence (Figure 2a).

To confirm the functionality of the FRET‐cubosomes, emission spectra were collected for intact cubosomes and for cubosomes treated with Triton X‐100, a detergent that disintegrates lipid‐based cubosomes. Both samples were excited at 460 nm, and emission spectra were measured using a microplate reader (Figure 2b). For intact cubosomes, the NBD‐PE emission peak was significantly suppressed, while a strong emission peak was observed at ≈585 nm for L‐Rhod‐PE, consistent with active FRET (Figure 2b). In contrast, Triton X‐100 treated cubosomes showed a reduced L‐Rhod‐PE peak and an increased NBD‐PE emission compared to intact cubosomes. These results confirmed the FRET mechanism in the cubosomes.

FRET‐cubosomes were then used to treat live CHO cells, and their interactions were imaged using fluorescence confocal microscopy over a 1 h period, capturing images every 5 min (Figure 2c). Untreated cells at 0 min served as controls. Initially, intact cubosomes appeared as bright red punctate spots in the L‐Rhod‐PE filter, likely representing the functional FRET pairs in free‐floating cubosomes or those wholly endocytosed and intact within cells. Over time, the NBD‐PE signal (green fluorescence) increased across the cells, peaking after 1 h, indicating that the FRET pair within the cubosomes became progressively “diluted” as cubosomes continuously fused with the CHO cell membranes (Figure 2c,d). A time‐lapse video of cubosome interactions with CHO cells over a 2 h period is provided in Movie S1 of the Supporting Information. To quantify these observations, the confocal images were analyzed in FIJI to measure the normalized fluorescence intensity in cells for both L‐Rhod‐PE and NBD‐PE signals (Figure 2d). The NBD‐PE signal is observed to progressively increase with time, while the L‐Rhod‐PE intensity remains at a constant level before slowly increasing after 30 min (Figure 2d), likely from the saturation of FRET‐cubosome lipids in the plasma membrane. The normalized intensities were then used to calculate the FRET efficiency over the 1 h period using the equation

(1) $$\mathrm{FRET}\;\mathrm{efficiency}=\frac{L-\mathrm{Rhod}-\mathrm{PE}\;\mathrm{intensity}}{L-\mathrm{Rhod}-\mathrm{PE}\;\mathrm{intensity}+\mathrm{NBD}-\mathrm{PE}\;\mathrm{intensity}}$$

The analysis revealed a progressively decreasing FRET efficiency (Figure 2e), consistent with the dilution of cubosome lipids into the cell membrane and increasing NBD‐PE intensity. During analysis, a threshold was applied to exclude the bright punctate fluorescence of intact cubosomes, ensuring the focus remained on the overall intracellular fluorescence. This step was critical to avoid random spikes in L‐Rhod‐PE fluorescence caused by free‐floating cubosomes in the cell media, which could otherwise lead to inconsistent intensity values.

**Figure 3:**
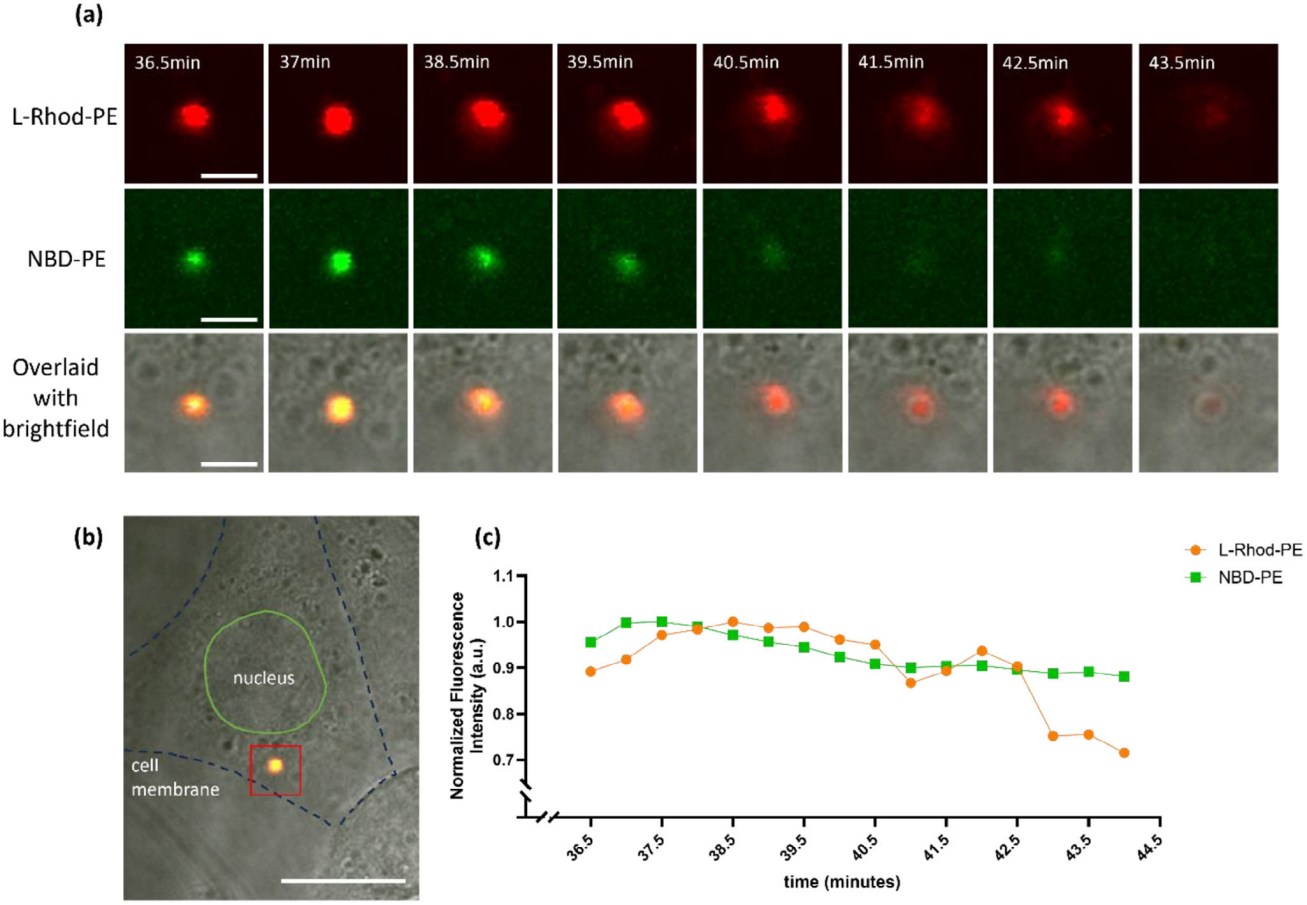
a) Confocal images from live cell‐imaging of a single endocytic event with a lifetime of ≈8 min from *t* = 36.5 min to *t* = 43.5 min. Scale bar = 5 µm. b) The whole cell where the single endocytic event was captured in the cell cytosol. Scale bar = 25 µm. c) Normalized fluorescence intensity plots of L‐Rhod‐PE and NBD‐PE of the single endocytic event across 8 min.

To confirm the FRET mechanism in cells, a control experiment was conducted where FRET‐cubosome‐treated cells were excited using both 460 nm (NBD‐PE) and 561 nm (L‐Rhod‐PE) lasers (Figure S5, Supporting Information). In intact FRET‐active cubosomes, where the FRET pair remains in close proximity, energy transfer from NBD‐PE to L‐Rhod‐PE occurs, leading to predominantly red fluorescence only in the FRET‐active cubosomes when only the NBD‐PE excitation laser is used (Figure S5, Supporting Information). However, when both lasers are used, the significantly increased brightness of L‐Rhod‐PE fluorescence confirmed the presence of L‐Rhod‐PE and NBD‐PE lipids distributed across the cells, indicating the saturation of the FRET‐inactive cubosome lipids that had fused with and integrated into the plasma membrane (Figure S5, Supporting Information). Importantly, the fluorescence changes observed in Figure 2 are unlikely to result from endocytic degradation. Endocytic degradation would typically cause either a loss of fluorescence signals due to breakdown or confinement of fluorescence within vesicular structures, as observed and explained later in **Figure** **3**. In contrast, the progressive dilution of fluorescence across the plasma membrane strongly supports the lipid mixing mechanism of cubosomes via membrane fusion.

Unlike endocytosis, a regulated cellular process,^[^ ^28^, ^29^ ^]^ much less is known about the regulation of LNP–cell membrane fusion. While membrane fusion is a well‐regulated process in natural biological systems, such as neurotransmission where proteins like SNARE complexes orchestrate synaptic vesicle fusion with the cell membrane,^[^ ^30^ ^]^ it remains unclear whether the fusion of LNPs, such as cubosomes, with cell membranes is a regulated process or merely a physical interaction.

Several studies suggest that the membrane fusion of cubosomes is largely driven by the physical properties of the LNPs, including their lipid composition^[^ ^31^, ^32^ ^]^ or nanostructure,^[^ ^9^, ^33^ ^]^ rather than intrinsic cellular mechanisms. The enhanced fusogenic behavior of cubosomes may be attributed to their unique physical properties, including high negative Gaussian curvature, which has been theorized to promote membrane fusion by lowering the energy barrier for fusion intermediate formation.^[^ ^34^, ^35^ ^]^ Prior studies have shown that the Gaussian curvature modulus ($\bar{\kappa }$) strongly influences fusogenicity, with cubosomes exhibiting a favorable curvature profile for fusion compared to liposomes or hexosomes.^[^ ^9^ ^]^ Additional factors such as lipid packing frustration and elastic deformation at the membrane interface may also facilitate fusion with cellular membranes.^[^ ^36^ ^]^

This contrasts with biological membrane fusion, which relies on tightly controlled protein‐mediated pathways. Our observations support this hypothesis, as the progressive increase in NBD‐PE fluorescence over time indicates continuous, unregulated fusion of cubosomes with the cell membrane (Figure 2c,d). This suggests that cubosome–cell membrane fusion is governed primarily by biophysical interactions rather than cellular regulatory processes. When cells were continuously saturated with cubosomes for up to 2 h (Movie S1, Supporting Information), both the NBD‐PE and L‐Rhod‐PE intensities continued to increase until the cell membrane became saturated with fluorescent lipids, eventually leading to cell death. This observation is consistent with findings by Strachan et al., who studied the cytotoxicity of cubosomes and liposomes in STO fibroblast cells.^[^ ^37^ ^]^ They reported that LNP induced toxicity is largely influenced by their interactions with the cellular membrane and internal organelles, particularly in uptake mechanisms involving membrane fusion. The oversaturation of nanoparticle lipids in the cell membrane can ultimately lead to cytotoxic effects.^[^ ^37^ ^]^

In our previous study, we similarly observed that liposomes were significantly less toxic than cubosomes.^[^ ^19^, ^25^ ^]^ We believe this difference is due, in part, to their cellular uptake mechanisms. Liposomes are more likely to be internalized through endocytosis,^[^ ^38^, ^39^, ^40^ ^]^ a regulated process, whereas cubosome uptake via membrane fusion appears unregulated. This unregulated fusion, while advantageous for applications requiring rapid delivery of contents, emphasizes the importance of carefully optimizing LNP formulations or delivery to mitigate potential cytotoxicity during prolonged cellular interactions.

### 2.3 Single‐Particle Tracking Reveals Endocytic Processes and Fluorescence Intensity Decay

To further investigate cubosome–cell interactions, live cell imaging was performed with shorter acquisition intervals, capturing images every 30 s to track single‐particle events. This approach enabled the observation of endocytic processes and provided insights into how endocytic fluorescence intensity changes differ from those associated with membrane fusion. FRET‐cubosomes, excited at 460 nm (NBD‐PE excitation only), were used to monitor the dynamic behaviors of individual particles within cells.

Figure 3a depicts a single cubosome event lasting ≈8 min, observed in the cytosol of the cell shown in Figure 3b. The particle appears as a bright punctate spot with both L‐Rhod‐PE and NBD‐PE fluorescence when excited at 460 nm (Figure 3a). In an FRET‐active cubosome, excitation at 460 nm (NBD‐PE) should result in energy transfer to L‐Rhod‐PE, producing a dominant red fluorescence signal. However, if FRET efficiency decreases, such as due to increased distance between fluorophores, NBD‐PE fluorescence becomes more prominent, indicating that the FRET interaction is weakening. The presence of L‐Rhod‐PE fluorescence in Figure 3a suggests that FRET is at least partially active and that the cubosome remains intact within the intracellular vesicle. However, at the same time, the presence of NBD‐PE fluorescence within the vesicle suggests that some lipid dilution is occurring, but only in the confined space of the vesicle (Figure 3a), rather than being freely dispersed throughout the plasma membrane as observed in membrane fusion (Figure 2c).

Brightfield imaging confirmed that the particle was confined within a vesicle during the event (e.g., Figure 3a, *t* = 41.5–43.5 min). At *t* = 36.5 min, the bright punctate spot measured ≈3 µm in diameter, consistent with macropinocytosis vesicle sizes.^[^ ^23^ ^]^ Over time, both NBD‐PE and L‐Rhod‐PE fluorescence steadily decreased until the signal was completely lost (Figure 3a), likely due to gradual degradation or dilution of fluorescent lipids within the vesicle. The normalized fluorescence intensities for both fluorophores were quantified using FIJI and plotted (Figure 3c), illustrating how the intensities of both fluorophores decayed steadily at similar rates. This behavior is distinctly different from membrane fusion events, where NBD‐PE fluorescence increases as the FRET pair dilutes (Figure 2d). When observing the L‐Rhod‐PE fluorescence (Figure 3a, top row), the particle initially appears as a bright punctate spot (*t* = 36.5–37 min). Over the next few minutes (*t* = 38.5–42.5 min), the particle begins to lose its punctate appearance, and fluorescence spreads within a limited circular region around the particle. At *t* = 43.5 min, the particle no longer appears punctate, leaving only faint, diffuse fluorescence.

The simultaneous decay of both fluorophores suggests that the observed event (Figure 3a,c) follows an endocytic pathway rather than FRET‐dilution associated with membrane fusion (Figure 2c). The fluorescence intensity changes of two additional endocytic events were analyzed, displaying similar L‐Rhod‐PE decay as the cubosomes are processed within endosomes (Figure S6, Supporting Information). This behavior aligns with a study tracking clathrin mediated endocytosis of liposomes in SUM159 cells, where fluorescence intensity decay indicated successful endocytic internalization, with endocytic event lifetimes ranging from 10 to 180 s.^[^ ^41^ ^]^ However, as the study focused solely on clathrin mediated pathways, the duration and involvement of other endocytic mechanisms, which may vary by cell type, remain unknown. Conversely, membrane fusion timelines in fibroblast and small intestine cells observed using TIRF microscopy ranged from 3 to 30 s,^[^ ^7^ ^]^ substantially shorter than the event observed in Figure 3.

### 2.4 TEM Reveals Cubosome Fusion with Plasma Membranes

**Figure 4:**
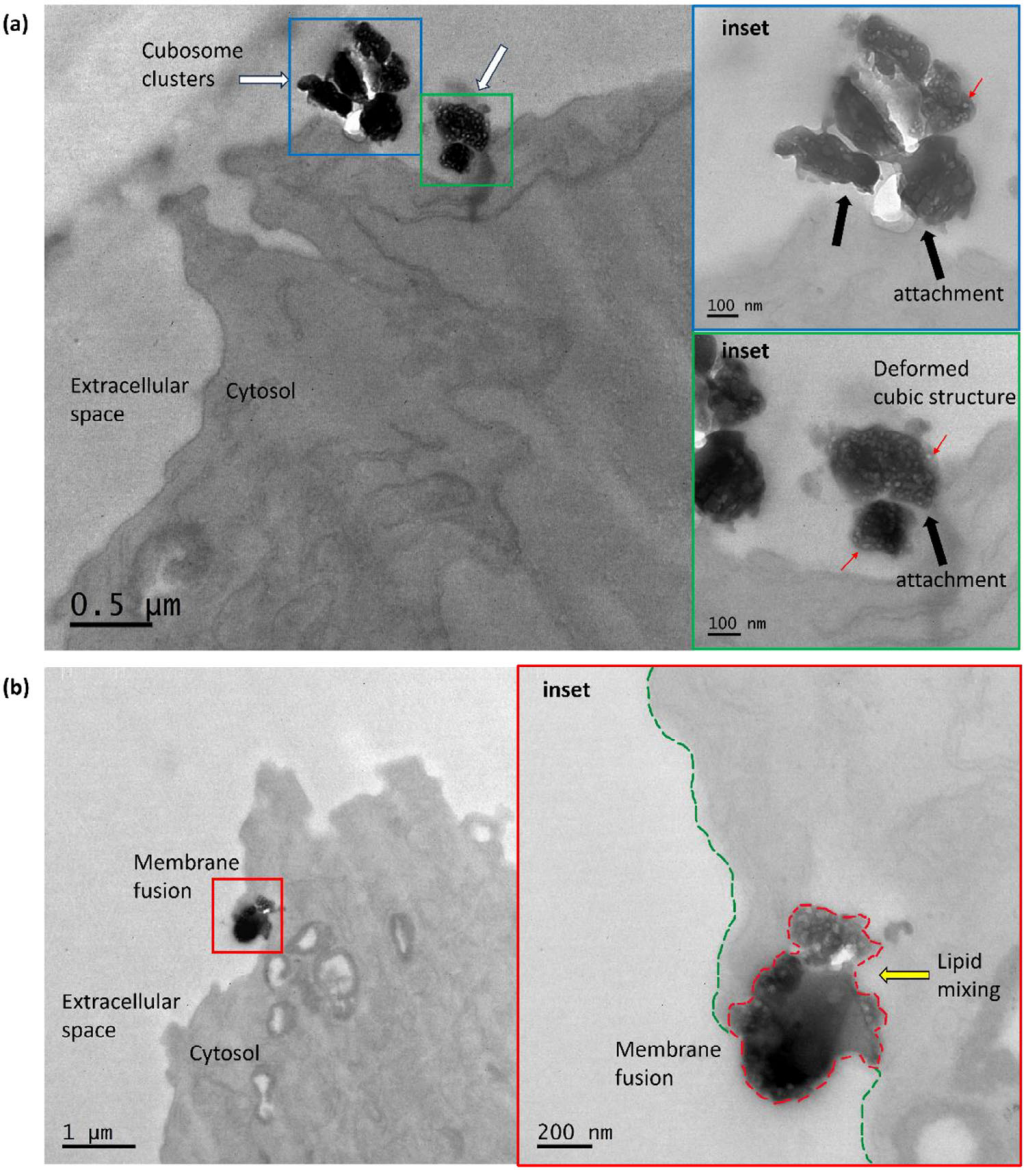
TEM images of cubosome‐treated CHO cells. a) Clustering behavior of cubosomes at an area on the cell membrane (white arrows). The blue and green insets provide a magnified view of each cubosome cluster. The porous structure of the internal cubic mesophase is observed (red arrows) in the insets, however, not as clearly defined due to the chemical processing during sample preparation. The cubosome clusters are observed to attach to the cell membrane surface (black arrows). b) Membrane fusion of cubosome with CHO cell plasma membrane. The red inset provides a magnified view of membrane fusion where the cubosome is embedded deep in the cell membrane and cytosol, illustrating a gradient as the cubosome lipids mixes (yellow arrow) with the plasma membrane and cell contents.

To observe cubosome–cell interactions at the nanometer scale, cubosome‐treated cells were fixed, stained, dehydrated, resin‐infiltrated, and sectioned into thin slices using an ultramicrotome before TEM imaging. Osmium tetroxide (OsO_4_), a chemical compound commonly used to stain lipid structures for electron microscopy, was used in the staining process of cubosome‐treated cells. OsO_4_ reacts with the unsaturated bonds in lipids, increasing electron density and enhancing the contrast of lipid rich structures.^[^ ^42^ ^]^ While the lipid composition of mammalian cell membranes may vary between cell types, ≈50% of the plasma membrane is composed of lipids,^[^ ^43^, ^44^ ^]^ as compared to the lipid dense cubosomes, which consists of 90 wt% Monoolein, an unsaturated lipid. This higher unsaturated lipid density in cubosomes results in disproportionate staining by OsO₄, making cubosomes appear darker than cells in TEM images due to their increased electron density (**Figure** **4**). Furthermore, all darker structures observed in the TEM images were between 200 and 500 nm in size, consistent with the typical size range of lipid‐based cubosomes. While chemically processing cells with paraformaldehyde, glutaraldehyde, OsO₄, and subsequent dehydration do not preserve the native hydrated nanostructure of cubosomes, the TEM images of cubosome‐treated cells will provide valuable insight into their interactions with cellular membranes and intracellular localization.

CHO cells were treated with R18‐cubosomes for 1 h. Clustering behavior of cubosomes was observed at the cell membrane surface (Figure 4a), where they appeared to closely interact with or anchor to the cell membrane. Given the multiple washing steps during sample preparation, it is unlikely that free‐floating cubosomes remained in the thin sections imaged, suggesting that the observed cubosome clusters are strongly associated with the cell membrane.

The membrane fusion behavior of cubosomes was also captured, with cubosomes embedded deeply within the lipidic cell membrane and cytosol (Figure 4b). This embedding was characterized by a gradient in staining intensity, with the external portion of the cubosome appearing circular and well‐defined, while the embedded portion progressively diffused into the cell. Notably, no membrane extensions/ruffles or vesicular membrane was observed wrapping around the cubosomes, which are characteristic features of endocytosis. The absence of such structures further supports the hypothesis that cubosomes can interact with cells via membrane fusion rather than only being internalized through endocytic pathways. These observations indicate that cubosomes can undergo a lipid exchange process during membrane fusion, where the cubosome lipids “bleed” into the cell membrane and cytosol (Figure 4b). The characteristic highly ordered bicontinuous cubic water channels (Figure 1a) are not observable in Figure 4, but instead we see heterogeneity in shading within the area of the imaged cubosomes, resembling pores scattered throughout the cubosome (Figure 4a, inset, red arrows).

### 2.5 Endosomal Interactions and Lipid Exchange of Au_10_NP‐Loaded Cubosomes

To investigate the intracellular behavior of cubosomes, CHO cells were treated with cubosomes containing 2% thiol‐coated gold nanoparticles less than 10 nm in diameter (Au_10_NP‐cubosomes), with an average diameter of ≈4 nm (Table S1, Supporting Information). The encapsulation of Au_10_NPs in cubosomes served two purposes: to study the intracellular distribution of cubosome cargo, and to enhance the contrast of cubosomes in TEM imaging by leveraging the high electron density of gold for easier detection. Au_10_NP‐cubosomes displayed a combination of *Im3m* (√2, √4, √6) cubic phase and weak hexagonal (√1, √3, √4) reflections,^[^ ^17^ ^]^ with an average hydrodynamic diameter and zeta potential of 200 nm and −2.79 mV, respectively (Figure S1b–e, Supporting Information). The weak hexagonal reflections are due to the occasional disruption of the mesophase structure at high Au_10_NP concentrations in some particles, as confirmed by cryo‐TEM (Figure S3a, Supporting Information). The Au_10_NPs are observed to integrate both in the water channels of cubosomes and the lipid bilayers (Figures S1a and S3a, Supporting Information). The relative electron density contrast images (color scaled from cryo‐TEM images) confirmed the presence of Au_10_NPs within the cubosomes, with Au_10_NPs appearing denser than the surrounding lipid matrix (Figure S7, Supporting Information). Cytotoxicity results of Au_10_NP‐cubosomes and size characterization of thiol‐capped Au_10_NPs can be found in the Supporting Information (Figures S4b and S8, Supporting Information).

**Figure 5:**
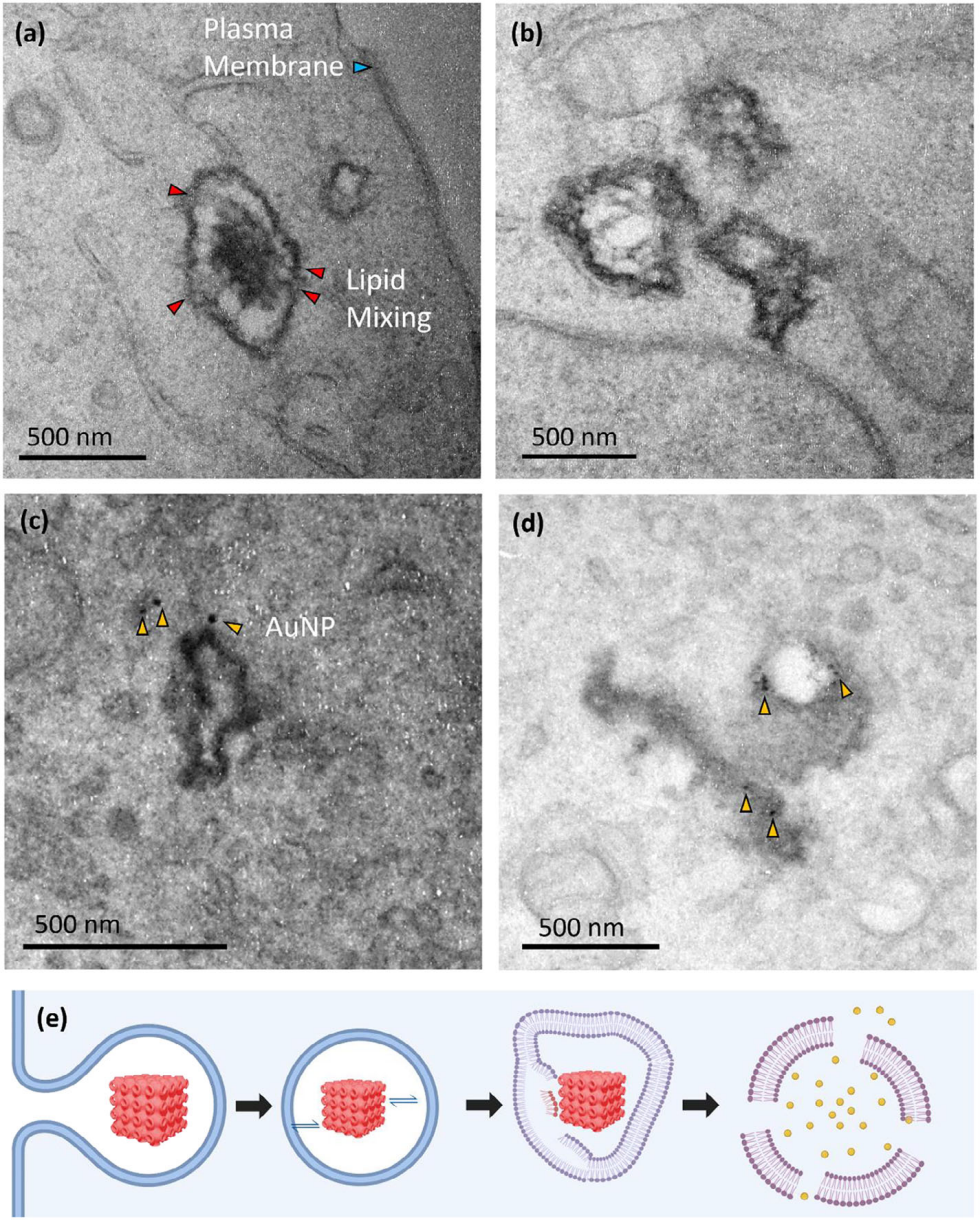
TEM images of Au_10_NP‐cubosome‐treated CHO cells. a) Cubosome residing within an endosome, where attachments (red arrows) between the cubosome and endosomal membrane indicating lipid mixing occurring. b) Disrupted endosome‐like structures. The dark electron dense structures indicate a lipid rich environment. c,d) Au_10_NPs in close proximity to the disrupted endosome structures, indicating the endosomal escape of Au_10_NP‐cubosomes. e) Illustration of the proposed process of how cubosomes disrupt the endosomal membrane via membrane fusion and lipid mixing, leading to the endosomal escape of cubosome cargo.

After 1 h of treatment, TEM images revealed an electron‐dense cubosome (≈250 and 470 nm in its shortest and longest diameters, respectively, consistent with the typical cubosome size range) residing within an early endosome upon internalization (**Figure** **5**a). This endosome appeared distinctly different from those observed in untreated control cells (Figure S9, Supporting Information). Close interactions between the cubosome and the endosome were observed, with multiple attachment points indicative of lipid mixing (Figure 5a, red arrows).

Several disrupted endosomal‐like structures are also observed (Figure 5b), lacking the smooth, round morphology typical of intact endosomes imaged in untreated control CHO cells (Figure S9, Supporting Information). These structural changes may reflect interactions between cubosomes and endosomal membranes, potentially involving lipid exchange processes that alter endosomal integrity. This is supported by the presence of free Au_10_NPs in the cytosol (Figure 5c,d, gold arrows). These free Au_10_NPs were located adjacent to disrupted endosomal remnants, suggesting that they originated from ruptured endosomes rather than direct fusion of cubosomes with the plasma membrane. Energy dispersive X‐ray spectroscopy (EDS) was performed on Au_10_NP‐cubosome‐treated cells during imaging to confirm the presence of gold in the cytosol (Figure S10, Supporting Information). These TEM observations of intracellular escape may help explain the increased intracellular fluorescence observed in cubosome‐treated cells compared to liposome‐treated cells (Figure 1), where fluorescence remains largely confined to endocytic compartments. Taken together, these findings support a model in which endocytosed cubosomes interact with and gradually destabilize endosomal membranes, potentially facilitating the release of encapsulated cargo into the cytosol. A schematic representation of this proposed process is illustrated in Figure 5e. While these findings offer high‐resolution structural insight into the process of endosomal disruption and cytosolic release, we acknowledge that TEM provides only static snapshots and cannot capture dynamic escape events in real time. Complementary live‐cell imaging techniques using pH‐sensitive or fluorescent cargo will be valuable in future studies to functionally validate and quantify endosomal escape kinetics.

Lipid exchange via membrane fusion between cubosomes and lipid membranes, particularly model membranes, has been well documented.^[^ ^45^, ^46^, ^47^ ^]^ In one study, cubosomes interacting with a POPC‐based lipid bilayer demonstrated an interfacial lipid exchange mechanism, where POPC molecules from the bilayer were exchanged with phytantriol molecules from the cubosomes.^[^ ^45^ ^]^ We propose that a similar lipid exchange phenomenon occurs between the cubosomes and endosomal membranes in our study (Figure 5), facilitating continuous lipid mixing and eventual endosomal destabilization.

Nanometer scale imaging of cubosome interactions has been explored in other studies using cryo‐EM and TEM. One study visualized antimicrobial peptide‐loaded cubosomes interacting with the outer bacterial membrane of mini *E. coli* cells using cryo‐EM.^[^ ^48^ ^]^ This imaging captured the initial stages of membrane fusion, where cubosomes attached to the outer bacterial membrane appeared to form a fusion pore without fully embedding into the cell. This behavior aligns with the early cubosome attachment we observed in our study (Figure 4a). Another study used TEM to investigate cubosome interactions with mammalian cells by incorporating gold‐labeled BSA into cubosomes.^[^ ^8^ ^]^ After 5 min of treatment, gold‐labeled BSA delivered via cubosomes was observed free in the cytosol, unlike free BSA‐gold, which was restricted to endosomes. Combined with our findings, these observations suggest that cubosomes can bypass or circumvent traditional endocytic pathways, whether through direct plasma membrane fusion or fusion with endosomal membranes.

### 2.6 Scanning Electron Microscopy Reveals Unique Surface Features of Cubosome‐Treated Cells

Following the observations of cubosome–plasma membrane fusion in TEM studies, we investigated whether the surface features of cubosome‐treated cells, as visualized by SEM, could provide additional evidence supporting direct plasma membrane fusion events. CHO cells were treated with R18‐cubosomes and compared to untreated control cells using SEM. In this study, 100 nm‐AuNPs (commercially sourced), which are internalized via endocytosis,^[^ ^49^, ^50^ ^]^ were also used for comparison. The 100 nm‐AuNPs should manifest as surface pits or invaginations during their endocytic uptake process.

**Figure 6:**
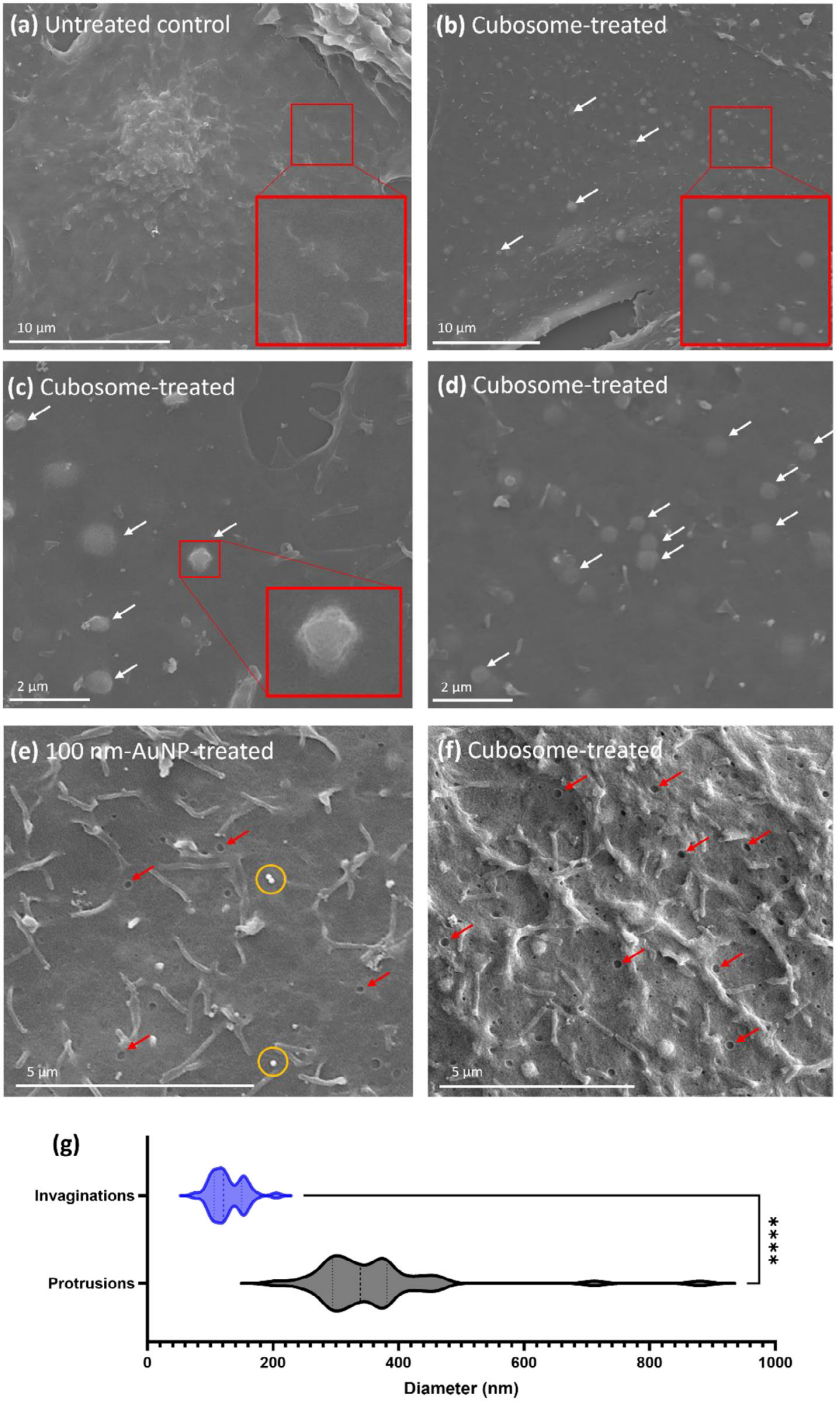
SEM images of CHO cell surface from different treatment conditions: a) untreated control, b–f) cubosome‐treated cells, and e) 100 nm‐AuNP treated cells. White arrows represent the circular protrusions/bumps found only on cubosome‐treated cells; red arrows represent invaginations/pits found on the cell surface of all treatment conditions, with a much higher frequency on the 100 nm‐AuNP treated and cubosome treated cells; and yellow circles represent the 100 nm‐AuNPs on the cell surface. g) Violin plot comparing the diameters of membrane surface protrusions (*n* = 48) and invaginations (*n* = 37) observed in SEM images of cubosome‐treated CHO cells. The plot displays the distribution, with median and interquartile ranges marked (dashed lines). Statistical significance was assessed using an unpaired two‐tailed *t*‐test (*p* < 0.05).

Cubosome‐treated cells displayed heterogeneous surface features, including circular protrusions, tubular extensions, and surface invaginations/pits (**Figure** **6**). Circular protrusions, unique to cubosome‐treated cells, were absent in untreated control and 100 nm‐AuNP‐treated cells (Figure 6a–e). These circular protrusions averaged 361 nm in diameter, ranging from 205 to 880 nm, based on 48 measurements across multiple cells. Similar surface asperities have been observed by Dyett et al. in bacteria treated with cubosomes, with spherical protrusions ranging from 50 to 400 nm.^[^ ^51^ ^]^ Interestingly, Dyett et al. observed relatively high contact angles of cubosomes at the bacteria surface (>90°), appearing as pronounced spherical protrusions.^[^ ^51^ ^]^ While bacterial studies revealed high contact angles^[^ ^48^, ^51^ ^]^ indicative of shallow attachment, or the initial stages of membrane fusion, the circular protrusions observed in CHO cells appeared flatter with smooth transitions into the plasma membrane (Figure 6b–d). Some instances of more pronounced protrusions can also be observed (Figure 6c, inset). The varying degree of protrusions observed likely represent different stages of the fusion process, with flatter protrusions indicating later stages. Unlike bacterial membranes, mammalian cells lack the additional carbohydrate barrier,^[^ ^51^ ^]^ such as peptidoglycan, which may enable more efficient membrane fusion and lipid exchange, exposing the cell cytosol to the cubosome cargo for the transfer of payloads upon membrane fusion. Currently the imaging of cubosomes with mammalian cell plasma membranes at the nanometer scale is nonexistent, with only limited studies of cubosomes and bacteria interactions at the nanometer scale.

Invaginations were also observed on both cubosome‐treated and 100 nm‐AuNP‐treated cells, as well as on untreated control cells, but were significantly more prevalent in treated cells (Figure 6, red arrows). The average diameters of these invaginations were 127 nm for cubosome‐treated cells and 121 nm for 100 nm‐AuNP‐treated cells. A previous study has reported similar observations of indentations on the surface of Cos‐7 cells, ranging from 50 to 160 nm in diameter, suggesting the presentation of clathrin‐coated or caveolar endocytic pits.^[^ ^52^ ^]^ Similar endocytic invaginations have been observed using SEM in single cell parasites such as *T. vaginalis* ^[^ ^53^ ^]^ and *Trypanosoma cruzi*.^[^ ^54^, ^55^, ^56^ ^]^ Tubular extensions, likely representing filopodia or microvilli, were also observed across all cell groups regardless of treatment (Figure 6), structures that are commonly present on cell surfaces.^[^ ^57^ ^]^

In our previous work, we reported the heterogeneous internalization mechanisms of cubosomes, with the presence of both endocytic uptake (primarily macropinocytosis) and passive nonendocytic internalization mechanisms, likely membrane fusion.^[^ ^19^ ^]^ Similarly, here we observe diverse surface features, with circular protrusions indicating membrane fusion and invaginations representing endocytic uptake mechanisms (Figure 6b–f). The invaginations observed here (averaging ≈120 nm, ranging from ≈76 to 205 nm in diameter) likely represent an array of endocytic vesicles such as clathrin‐coated pits of caveolae,^[^ ^52^ ^]^ consistent with the internalization mechanisms elucidated in our previous study.^[^ ^19^ ^]^

Based on the size measurements of both TEM and SEM experiments, we herein propose that larger cubosomes, which exceed the typical endosomal vesicle limit of most endocytic pathways (excluding that of macropinocytosis and phagocytosis) are more conducive to membrane fusion in mammalian cells, while smaller cubosomes or coexisting vesicles in cubosome formulations are more likely to be internalized via endocytic mechanisms. This is supported by the observed size range of the circular protrusions (≈205–880 nm), indicating the uptake of larger cubosomes surpassing ≈200 nm is primarily limited to membrane fusion or macropinocytosis internalization pathways. However, as demonstrated by the endosomal escape of Au_10_NP‐loaded cubosomes in TEM studies (Figure 5), endocytic internalization does not necessarily hinder cubosomes or lead to their recycling or lysosomal degradation. Instead, cubosomes can fuse with and destabilize endosomal membranes, facilitating escape.

The ideal nanoparticle size for bioactive cargo delivery can vary based on the targeted application. Clinically approved LNPs, such as those used in COVID‐19 mRNA vaccines, vary in size depending on formulation. For instance, Pfizer's Comirnaty particles exhibit hydrodynamic diameters ≈60–90 nm, while Moderna's Spikevax includes a broader size distribution, with the main population ranging from 100 to 200 nm and an average diameter of ≈217 nm.^[^ ^58^ ^]^ In contrast, the cubosomes in this study exhibited average hydrodynamic diameters between 200 and 250 nm, placing them at the upper end of the LNP size spectrum.

Nanoparticle size is a key determinant of in vivo biodistribution, immune response, and organ tropism.^[^ ^59^, ^60^ ^]^ For example, LNPs ranging from 60 to 150 nm have been reported to be efficient for vaccine delivery as they evoke robust immune responses essential for the recruitment of immune cells, or antigen presenting cells for antigen expression;^[^ ^61^ ^]^ whereas exosome‐like nanoparticles ranging from 40 to 50 nm were revealed to be the most effective in siRNA delivery resulting in gene silencing, as compared to NPs ranging 100–200 nm.^[^ ^62^ ^]^ Particles smaller than 100 nm have been shown to more readily extravasate from injection sites, drain via lymphatics, and accumulate in organs such as the liver and spleen.^[^ ^60^ ^]^ However, size alone does not dictate delivery efficacy. In rodent models, mid‐sized LNPs (≈180 nm) resulted in higher mRNA expression in the liver than smaller (≈80 nm) or larger (≈330 nm) particles, suggesting differences in cellular uptake and intracellular trafficking pathways.^[^ ^60^ ^]^ Larger particles (≈300–330 nm) tended to remain near the injection site and showed enhanced accumulation in local tissues but lower systemic gene expression.^[^ ^60^ ^]^

However, LNP delivery efficacy is still limited by endocytic recycling and dependent on the endosomal escape of LNPs into the cytosol.^[^ ^5^, ^6^ ^]^ Thus, while the larger size of cubosomes may result in a distinct biodistribution profile compared to sub‐100 nm LNPs, their membrane fusion capability may compensate for limited systemic access by enhancing direct cytosolic delivery and bypassing endosomal entrapment. Furthermore, tuning cubosome size may influence their uptake mechanism—larger cubosomes may favor membrane fusion or macropinocytosis, while smaller cubosomes may be internalized via clathrin‐mediated endocytosis.^[^ ^19^ ^]^ Future studies will be needed to identify the optimal size range of cubosomes for different delivery routes and therapeutic applications.

**Figure 7:**
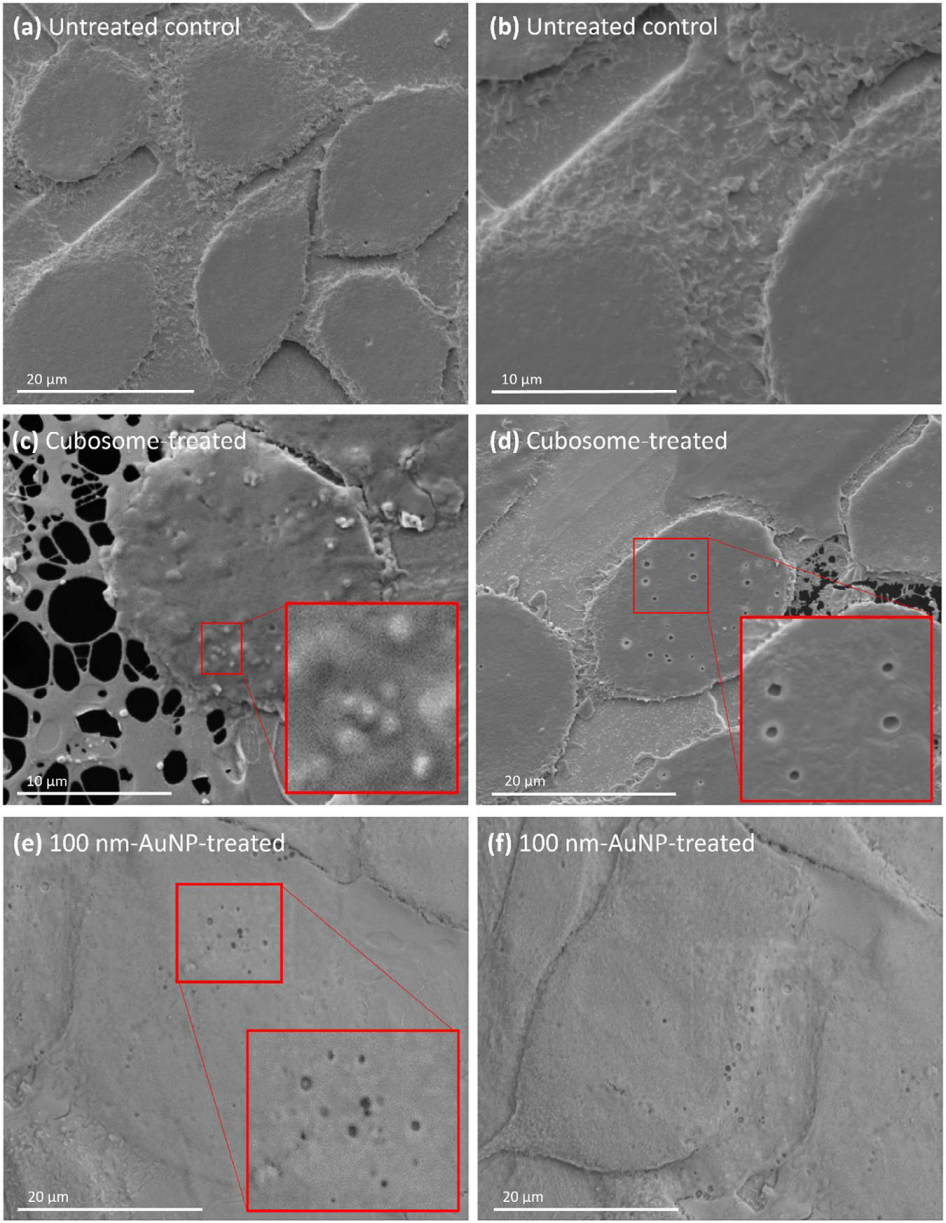
Cryo‐SEM images of CHO cell surface from different treatment conditions. a,b) untreated cells, c,d) cubosome treated cells, and e,f) 100 nm‐AuNP treated cells. The insets in cubosome treated cells highlight circular protrusions and invaginations on the cell surface. The red inset in 100 nm‐AuNP treated cells highlight invaginations on the cell surface from 100 nm‐AuNP treatment.

Cryo‐SEM was used to further validate the surface features observed in SEM and to study cubosome–cell interactions in their frozen hydrated state (without chemical treatment). After 1 h of cubosome treatment, cells were washed, lightly blotted, plunge‐frozen in liquid nitrogen, subjected to ice sublimation, and sputter‐coated before imaging. Cryo‐SEM revealed similar circular protrusions and pores on the surface of cubosome‐treated cells (**Figure** **7**c,d), consistent with observations from conventional SEM (Figure 6). However, the circular protrusions imaged with cryo‐SEM appeared less pronounced and flatter compared to those observed with SEM (Figure 7c, inset). The size distribution of the protrusions matched that observed in SEM, while the pores appeared slightly smaller in cryo‐SEM images. Cryo‐SEM also showed smoother cell surfaces with fewer visible tubular extensions compared to the chemically processed and dehydrated cells imaged using SEM (Figure 7).

These findings align with a study by Noble et al., which compared the morphology of chemically fixed cells in conventional SEM and frozen hydrated cells in cryo‐SEM.^[^ ^57^ ^]^ In conventional SEM, cell surfaces appeared relatively flat with prominent tubular extensions, whereas cryo‐SEM preserved the cells’ rounded morphology with fewer obvious protrusions. The reduced visibility of tubular extensions in cryo‐SEM is likely due to the blotting step during sample preparation, which caused these extensions to collapse and lie flat against the grid.^[^ ^57^ ^]^ In contrast, the dehydration process in conventional SEM preserved 3D tubular projections and spherical structures but chemically altered the cells' native state. Similarly, we observed that protruding surface features, including circular protrusions and tubular extensions, appeared less pronounced in cryo‐SEM (Figure 7c, inset) than in SEM (Figure 6b–d). Control experiments with 100 nm‐AuNPs, internalized via endocytosis, showed similar surface pores on treated cells (Figure 7d.e), while untreated cells exhibited predominantly smooth surfaces with significantly fewer pores (Figure 7a,b). Conclusively, both SEM and cryo‐SEM consistently highlighted the key surface features of cubosome‐treated cells: unique circular protrusions and an increased number of invaginations.

## 3 Conclusion

This study comprehensively investigated cubosome interactions with mammalian cells using a suite of advanced microscopy techniques, including TEM, SEM, Cryo‐SEM, and live‐cell fluorescence imaging. Each method provided unique insights, enabling us to capture critical micro‐ and nanoscale events that shed light on the complex and distinctive behaviors of cubosomes in cellular environments.

Notably, we visualized, for the first time in mammalian cells, the membrane fusion of cubosomes with both the plasma and endosomal membranes at the nanoscale. This demonstrated their distinct ability to bypass or circumvent endocytic recycling and lysosomal degradation due to their unique physical properties, enabling lipid exchange and endosomal membrane disruption, which facilitates the escape of cubosome cargo into the cytosol. Additionally, we identified unique surface features, such as circular protrusions specific to cubosome treatment and invaginations indicative of endocytic activity, further emphasizing their diverse internalization mechanisms. At the microscale, fluorescently labeled cubosomes and FRET‐functionalized cubosomes revealed the localization of cubosome lipids along the plasma membrane and the progressive saturation of cubosome lipids in the plasma membrane, underscoring their membrane fusion capability. This contrasts sharply with the behavior of liposomes, which are primarily internalized via endocytosis.

While we successfully visualized cubosome membrane fusion with mammalian cell membranes, future advancements in imaging techniques, particularly those capable of capturing real‐time interactions in their native state, could provide deeper insights into the dynamics of fusion and internalization processes.

This study offers a foundational framework for understanding cubosome–cell interactions, opening new avenues for designing lipid‐based delivery systems. By directly visualizing the fusion capabilities of cubosomes and unraveling their mechanisms, this research highlights their potential for efficient cargo delivery directly into the cytosol. These insights may drive advancements in nanomedicine, particularly for applications in targeted drug delivery, gene therapy, and vaccine development, where precise and efficient intracellular delivery is essential.

## 4 Experimental Section

### Cubosome Formulation: Fluorescently Labeled Cubosomes

R18 labeled cubosomes were formulated using a microfluidic benchtop system, the NanoAssemblr (Precision NanoSystems). Monoolein (Nuchek Prep) was dissolved in ethanol at 100 mg mL^−1^. Pluronic‐F127 (Sigma‐Aldrich) was dissolved in a 1:1 solution of ethanol and methanol at 10 mg mL^−1^. R18 (Invitrogen) was dissolved in ethanol at 1 mg mL^−1^. The stock solutions were mixed and diluted with ethanol to form an input stock solution with a final concentration of 40, 4, and 0.04 mg mL^−1^ for Monoolein, Pluronic F‐127, and R18, respectively. A 1 mL syringe was filled with the stock solution containing the dissolved lipids, and a 3 mL syringe was filled with Milli‐Q water. The syringes were fitted into the cartridge on the NanoAssemblr and mixed at a flow ratio of 3:1 for the aqueous phase to organic phase, with a flow rate of 24 mL min^−1^ to produce 1 mL of nanoparticle formulation. The formulation was then left in a vacuum oven at 40 °C for 6 h to evaporate the organic solvent. The formulation method was selected based on achieving the lowest PDI for R18‐cubosomes.

### FRET‐Functionalized Cubosomes

FRET‐functionalized cubosomes were formulated by ultrasonication of a thin lipid film. For this, monoolein, oleyl acetate, Pluronic F‐127, L‐Rhod‐PE, and NBD‐PE were dissolved in ethanol at 100, 100, 20, 2, and 2 mg mL^−1^, respectively. With monoolein as the primary lipid, the fluorescent lipids L‐Rhod‐PE and NBD‐PE were mixed in at 0.25 mol% each, and Pluronic F‐127 at 10 wt%, and oleyl acetate at 5 wt%. The stock solutions were then mixed at appropriate quantities in a 1 mL Eppendorf tube and then left to dry in a vacuum oven overnight at 40 °C. The dried lipid film was then ultrasonicated with 1 mL of Milli‐Q water with an amplitude of 30, for 3 s on, 5 s off, with a final concentration of 10 mg mL^−1^. The formulation method was selected based on achieving the lowest PDI for FRET‐based cubosomes.

### 10 nm Gold Nanoparticle (Au_10_ NP)‐Loaded Cubosomes

Thiol‐coated gold nanoparticles of less than 10 nm in diameter (Au_10_NP) were formulated for loading in cubosomes. The formulation protocol of thiol‐coated Au_10_NP follows a protocol previously described by Brust et al.^[^ ^63^ ^]^ in detail. The only deviation from the protocol is that the final formulation of Au_10_NP was suspended in hexane instead of toluene. Briefly, AuCl_4_− was reduced by sodium borohydride in the presence of an alkanethiol using a two‐phase reduction process. The Au_10_NPs were finally suspended in hexane for solvent compatibility with monoolein. A stock solution of monoolein in hexane was prepared and then mixed with 2 wt% of the Au_10_NP solution. This mixture was left to dry in a vacuum oven overnight at 40 °C. The dried lipid film was then ultrasonicated with 1 mL of Pluronic F‐127 in Milli‐Q water at 10 wt%, with an amplitude of 30, for 3 s on, 5 s off, with a final concentration of 10 mg mL^−1^. The formulation method was selected due to solvent compatibility for the Au_10_NPs.

### Cubosome Physicochemical Characterization: Small Angle X‐Ray Scattering (SAXS)

SAXS was performed at the small and wide‐angle X‐ray scattering (SAXS/WAXS) beamline at the Australian Synchrotron operated by ANSTO. All measurements were performed at 25 °C unless stated otherwise. The beam wavelength was 1.032 Å with a typical flux of 1013 photon s^−1^. The sample to detector distance was set at 1.6 m, with a q‐range of 0.01–0.5 Å. The X‐ray diffraction patterns were recorded on a Dectris‐Pilatus 1 M detector. The scattering images were interpreted into 1D diffraction patterns using Axcess, an IDL‐based software, to investigate the mesostructure of the cubosomes. The concentration of cubosome solutions during SAXS measurements was 20 mg mL^−1^ in water and cell media.

### Dynamic Light Scattering

The hydrodynamic diameter, particle size distribution, and PDI were measured using the Nano ZS Zetasizer (Malvern Instruments). Cubosomes were diluted to 1 mg mL^−1^ in the Milli‐Q water and 200 µL of the solution was added to a 96 well plate for dynamic light scattering measurements on the Zetasizer. All measurements were performed at 25 °C unless stated otherwise.

### Zeta Potential Measurements

The zeta potential of cubosomes was measured using the Nano ZS Zetasizer (Malvern Instruments). Nanoparticles were diluted in 0.01× PBS to provide a low ionic strength solution for accurate measurements of zeta potential.^[^ ^25^ ^]^ 1 mL of diluted cubosomes was added to a disposable folded capillary and measured at 25 °C in the Zetasizer.

### Cryogenic Transmission Electron Microscopy (Cryo‐TEM)

The cubosomes were imaged in their native frozen hydrated state using cryo‐TEM. An automated plunge freezer, the FEI Vitrobot was used for cubosome vitrification. Lacey carbon‐coated copper grids were glow discharged to induce hydrophilicity on the carbon side of the grid. 4 µL of cubosome solution (at a minimum of 10 mg mL^−1^) was dripped onto the grid and vitrified in liquid ethane with the Vitrobot settings of blot force: 4, blot time: 4 s, and a humidity of 95%. The grids were then rapidly transferred to a Gatan 626 cryo‐holder or stored for later imaging. All samples were imaged using the FEI Tecnai F30 Transmission Electron Microscope operating at 200 kV at defocus levels between −4 and −6 µm.^[^ ^64^ ^]^

### Cell Culture

CHO epithelial cells were thawed from liquid nitrogen storage and incubated in a cell culture incubator at 37 °C and 5% CO_2_, with DMEM/F12 (Gibco) media supplemented with 10% fetal bovine serum (FBS) and 1% penicillin–streptomycin (Gibco). All further mentions of incubation in a cell culture incubator will assume the same conditions, unless stated otherwise. All CHO cells used in experiments maintained a passage number below 12. Cells were suspended using TrypLE Express Enzyme (1×) (Gibco) prior to cell seeding.

### Confocal Microscopy: Cell Uptake of R18‐Cubosomes

CHO cells were suspended and seeded at 20 000 cells per well in an 8 well confocal microscopy chamber slide and left to incubate in a cell culture incubator overnight. The cells were then washed twice with PBS and 200 µL and incubated in 25 µg mL^−1^ of nanoparticle solution diluted in serum‐free DMEM/F12 media. After 1 h of treatment, the cells were gently rinsed three times with PBS and fixed using a 4% formaldehyde solution for 30 min at room temperature. Lastly, the cells were rinsed twice with cold PBS and left in PBS solution for imaging. The cells were imaged using the Nikon Ti‐Eclipse Confocal Microscope, with the ×100 oil immersion lens. Cells were imaged under brightfield and TRITC laser/emission filter (561 nm/595 ± 25 nm).

### Cell Uptake of FRET‐Cubosomes

CHO cells were suspended and seeded at 20 000 cells per well in an 8 well confocal microscopy chamber slide and left to incubate in a cell culture incubator overnight. The cells were then washed twice with PBS and incubated in 100 µL serum‐free media in the confocal microscope chamber maintained at 37 °C and 5% CO_2_. The cells were imaged live using the Nikon Ti‐Eclipse Confocal Microscope, with the ×100 oil immersion lens, using brightfield, and FITC (525 ± 25 nm) and TRITC (595 ± 25 nm) emission filters. During FRET‐based experiments, only the FITC (488 nm) excitation laser was used. The untreated cells were imaged once (as a control) before 100 µL of FRET‐functionalized cubosomes diluted in serum‐free media at 25 µg mL^−1^ was added to the confocal microscope chamber and imaged for 1 h with an acquisition time of every 5 min. The experiment was then repeated with an acquisition time of every 30 s for the visualization of endocytic events.

### Transmission Electron Microscopy (TEM)

TEM samples were prepared using two methods—by preparation of cells on coverslips and by cell pellets.

For the coverslips, 18 mm coverslips were sterilized with 100% ethanol and then left under UV light exposure for 30 min. The sterile coverslips were then washed with PBS and placed in a 12 well plate. A cell suspension of 25 000 cells mL^−1^ was prepared and 1 mL of the cell suspension was seeded in a 12 well plate containing the coverslips and left to incubate overnight in a cell culture incubator. The cells were then washed three times with PBS and incubated in 25 µg mL^−1^ of cubosome solution diluted in serum‐free DMEM/F12 media for 1 h. The coverslips were then gently rinsed in 0.1 m cacodylate buffer and fixed in 2% paraformaldehyde and 2.5% glutaraldehyde in cacodylate buffer for 1 h at room temperature, shielded from light. The cells were then gently rinsed three times in 0.1 m cacodylate buffer and stained with 1% OsO_4_ and 1.5% potassium ferrocyanide diluted in distilled water for at least 90 min. The cells were rinsed three times with Milli‐Q water and dehydrated using an ethanol graded series from 75–100% and 100% acetone. The cells were infiltrated with Spurr's resin and polymerized in the oven at 60 °C for at least 48 h. The cells were then sliced using an ultramicrotome and poststained and imaged under the JEOL 1010 Transmission Electron Microscope.

For the cell pellet, CHO cells were seeded in a T175 cell culture flask and left to incubate until the cell confluency exceeded 90%. The cells were then washed three times with PBS and incubated in 25 µg mL^−1^ of cubosome solution diluted in serum‐free DMEM/F12 media for 1 h. The cell media was then aspirated and immediately replaced with 2% paraformaldehyde and 2.5% glutaraldehyde in 0.1 m cacodylate buffer for 1 h at room temperature, shielded from light. After fixation, the cells were gently scraped and collected in a tube. The cells were then centrifuged at 1000 rpm for 5 min to form a cell pellet. The supernatant was aspirated, and the cells were gently rinsed by resuspending the cells in 0.1 m cacodylate. This centrifuging and rinsing process was repeated twice, before finally suspending the cells in 1% OsO_4_ and 1.5% potassium ferrocyanide diluted in distilled water for at least 90 min. The cells were then centrifuged to remove the supernatant and resuspended three times in distilled water. The cells were then dehydrated using an ethanol graded series from 75–100% and 100% acetone via centrifugation and resuspension. The cells were finally resuspended in Spurr's resin and polymerized in the oven at 60 °C for at least 48 h. The cells were then sliced using an ultramicrotome, poststained and imaged under the JEOL 1010 Transmission Electron Microscope.

### Scanning Electron Microscopy (SEM)

25 mm coverslips were sterilized with 100% ethanol and then left under UV light exposure for 30 min. The sterile coverslips were then washed with PBS and placed in a 6 well plate. A cell suspension of 25 000 cells mL^−1^ was prepared, and 2 mL of the cell suspension was seeded in a 6 well plate containing the coverslips and left to incubate overnight in a cell culture incubator. The cells were then washed three times with PBS and incubated in 25 µg mL^−1^ of cubosome solution or 100 nm gold nanoparticle solution (Sigma‐Aldrich), diluted in serum‐free DMEM/F12 media, for 1 h. The cells were then gently rinsed in 0.1 m cacodylate buffer and fixed in 4% paraformaldehyde and 2.5% glutaraldehyde in cacodylate buffer for 30 min at room temperature, shielded from light. The cells were then gently rinsed three times in buffer and stained with 2% OsO_4_ and 1.5% potassium ferrocyanide for 1 h. The cells were rinsed three times with Milli‐Q water and incubated in 1% thiocarbohydrazide in distilled water for 20 min. The cells were then rinsed three times with Milli‐Q water and left to incubate in 2% OsO_4_for further staining. The cells were then rinsed three times with Milli‐Q water and dehydrated in an ice‐cold solution ethanol graded series from 50% to 100% and finally with 100% acetone. Finally, the cells were coated with 10 nm of iridium with Leica Sputter coater and imaged under the FEI Quanta 200 FEG Scanning Electron Microscope. The surface features were then measured using FIJI. 100 nm‐AuNP suspension was obtained from Sigma‐Aldrich. To quantitatively assess surface features observed in SEM images, the diameters of circular protrusions and invaginations were manually measured using ImageJ. Features were outlined using the elliptical selection tool across representative regions of cubosome‐treated cell surfaces where the membrane morphology was clearly resolvable.

### Cryogenic Scanning Electron Microscopy (Cryo‐SEM)

Carbon coated gold 200 mesh grids were sterilized with 100% ethanol and then left under UV light exposure for 30 min. The sterile grids were then washed with PBS and placed in a 6 well plate. A cell suspension of 25 000 cells mL^−1^ was prepared, and 2 mL of the cell suspension was seeded in a 6 well plate containing the grids and left to incubate overnight in a cell culture incubator. The cells were then washed three times with PBS and incubated in 25 µg mL^−1^ of cubosome solution diluted in serum‐free DMEM/F12 media for 1 h. The cells were then rinsed once with PBS, loaded onto a grid holder and immediately cryo‐immobilized with a liquid nitrogen plunger. The grids were then rapidly transferred to the preparation chamber on the FEI Quanta 200 FEG SEM equipped with a Gatan Alto Cryo‐SEM vacuum transfer device. The grids were heated from −150 °C to −100 °C for 20 min and heated to −90 °C for a further 90 s to facilitate ice sublimation and expose the cubosome‐treated cell surface.^[^ ^57^ ^]^ The grids were then transferred into the Quanta 200 FEG SEM preparation chamber at −150 °C and sputter coated with Au for 90 s.^[^ ^57^ ^]^ Finally, SEM images were acquired. The surface features were then measured using FIJI. 100 nm‐AuNP suspension was obtained from Sigma‐Aldrich.

### Statistical Analysis

Statistical comparisons of surface feature diameters in SEM images were performed using an unpaired two‐tailed *t*‐test, with significance set at *p* < 0.05.

## Conflict of Interest

The authors declare no conflict of interest.

## Supporting information



Supporting Information

Supplemental Movie 1

## Data Availability

The data that support the findings of this study are available from the corresponding author upon reasonable request.

## References

[1] A. Akinc , M. A. Maier , M. Manoharan , K. Fitzgerald , M. Jayaraman , S. Barros , S. Ansell , X. Du , M. J. Hope , T. D. Madden , B. L. Mui , S. C. Semple , Y. K. Tam , M. Ciufolini , D. Witzigmann , J. A. Kulkarni , R. van der Meel , P. R. Cullis , Nat. Nanotechnol. 2019, 14, 1084. 31802031 10.1038/s41565-019-0591-y

[2] J. Zhai , C. Fong , N. Tran , C. J. Drummond , ACS Nano 2019, 13, 6178. 31082192 10.1021/acsnano.8b07961

[3] U. Bulbake , S. Doppalapudi , N. Kommineni , W. Khan , Pharmaceutics 2017, 9, 12. 28346375 10.3390/pharmaceutics9020012 PMC5489929

[4] S. Behzadi , V. Serpooshan , W. Tao , M. A. Hamaly , M. Y. Alkawareek , E. C. Dreaden , D. Brown , A. M. Alkilany , O. C. Farokhzad , M. Mahmoudi , Chem. Soc. Rev. 2017, 46, 4218. 28585944 10.1039/c6cs00636a PMC5593313

[5] G. Sahay , W. Querbes , C. Alabi , A. Eltoukhy , S. Sarkar , C. Zurenko , E. Karagiannis , K. Love , D. Chen , R. Zoncu , Y. Buganim , A. Schroeder , R. Langer , D. G. Anderson , Nat. Biotechnol. 2013, 31, 653. 23792629 10.1038/nbt.2614 PMC3814166

[6] J. Gilleron , W. Querbes , A. Zeigerer , A. Borodovsky , G. Marsico , U. Schubert , K. Manygoats , S. Seifert , C. Andree , M. Stöter , H. Epstein‐Barash , L. Zhang , V. Koteliansky , K. Fitzgerald , E. Fava , M. Bickle , Y. Kalaidzidis , A. Akinc , M. Maier , M. Zerial , Nat. Biotechnol. 2013, 31, 638. 23792630 10.1038/nbt.2612

[7] B. P. Dyett , H. Yu , J. Strachan , C. J. Drummond , C. E. Conn , Nat. Commun. 2019, 10, 4492. 31582802 10.1038/s41467-019-12508-8 PMC6776645

[8] J. A. Prange , S. Aleandri , M. Komisarski , A. Luciani , A. Käch , C.‐D. Schuh , A. M. Hall , R. Mezzenga , O. Devuyst , E. M. Landau , ACS Appl. Bio Mater. 2019, 2, 2490. 10.1021/acsabm.9b00187 35030705

[9] L. Zheng , S. R. Bandara , Z. Tan , C. Leal , Proc. Natl. Acad. Sci. USA 2023, 120, 2301067120. 10.1073/pnas.2301067120 PMC10318962 37364130

[10] C. Leal , N. F. Bouxsein , K. K. Ewert , C. R. Safinya , J. Am. Chem. Soc. 2010, 132, 16841. 21028803 10.1021/ja1059763 PMC2991473

[11] H. Yu , B. P. Dyett , J. Zhai , J. B. Strachan , C. J. Drummond , C. E. Conn , J. Colloid Interface Sci. 2023, 634, 279. 36542965 10.1016/j.jcis.2022.12.028

[12] C. Oliveira , C. J. O. Ferreira , M. Sousa , J. L. Paris , R. Gaspar , B. F. B. Silva , J. A. Teixeira , P. Ferreira‐Santos , C. M. Botelho , Nanomaterials 2022, 12, 2224. 35808060 10.3390/nano12132224 PMC9268278

[13] L. Zhang , J. Li , D. Tian , L. Sun , X. Wang , M. Tian , Cell Death Dis. 2020, 11, 1. 31911576 10.1038/s41419-019-2182-0 PMC6946659

[14] S. Sarkar , N. Tran , S. K. Soni , Z. Nasa , C. J. Drummond , C. E. Conn , ACS Appl. Mater. Interfaces 2021, 13, 2336. 33410653 10.1021/acsami.0c20956

[15] S. Rajesh , S. Gangadoo , H. Nguyen , J. Zhai , C. Dekiwadia , C. J. Drummond , J. Chapman , V. K. Truong , N. Tran , ACS Appl. Mater. Interfaces 2022, 14, 32845. 35850116 10.1021/acsami.2c05165

[16] J. B. Strachan , B. P. Dyett , N. C. Jones , S. V. Hoffmann , C. Valery , C. E. Conn , J. Colloid Interface Sci. 2021, 592, 135. 33647562 10.1016/j.jcis.2021.02.027

[17] C. V. Kulkarni , W. Wachter , G. Iglesias‐Salto , S. Engelskirchen , S. Ahualli , Phys. Chem. Chem. Phys. 2011, 13, 3004. 21183976 10.1039/c0cp01539c

[18] Y. Huang , Z. Chang , X. Xia , Z. Zhao , X. Zhang , Z. Huang , C. Wu , X. Pan , J. Nanopart. Res. 2023, 25, 176.

[19] S. L. Yap , B. Dyett , A. J. Hobro , H. Nguyen , N. I. Smith , C. J. Drummond , C. E. Conn , N. Tran , Small 2025, 2500903. 40392028 10.1002/smll.202500903 PMC12508709

[20] E. Jabłonowska , D. Matyszewska , E. Nazaruk , M. Godlewska , D. Gaweł , R. Bilewicz , Biochim. Biophys. Acta, Gen. Subj. 2021, 1865, 129738. 32956751 10.1016/j.bbagen.2020.129738

[21] H.‐H. Shen , V. Lake , A. P. Le Brun , M. James , A. P. Duff , Y. Peng , K. M. McLean , P. G. Hartley , Biomaterials 2013, 34, 8361. 23899446 10.1016/j.biomaterials.2013.07.042

[22] M. Malatesta , Int. J. Mol. Sci. 2021, 22, 12789. 34884592 10.3390/ijms222312789 PMC8657944

[23] J. P. Lim , P. A. Gleeson , Immunol. Cell Biol. 2011, 89, 836. 21423264 10.1038/icb.2011.20

[24] J. Canton , Front. Immunol. 2018, 9, 2286. 30333835 10.3389/fimmu.2018.02286 PMC6176211

[25] S. L. Yap , H. Yu , S. Li , C. J. Drummond , C. E. Conn , N. Tran , J. Colloid Interface Sci. 2024, 656, 409. 38000253 10.1016/j.jcis.2023.11.059

[26] T. Chen , B. He , J. Tao , Y. He , H. Deng , X. Wang , Y. Zheng , Adv. Drug Delivery Rev. 2019, 143, 177. 10.1016/j.addr.2019.04.009 31201837

[27] J. Gravier , L. Sancey , S. Hirsjärvi , E. Rustique , C. Passirani , J.‐P. Benoît , J.‐L. Coll , I. Texier , Mol. Pharmaceutics 2014, 11, 3133. 10.1021/mp500329z 25098740

[28] G. J. Doherty , H. T. McMahon , Annu. Rev. Biochem. 2009, 78, 857. 19317650 10.1146/annurev.biochem.78.081307.110540

[29] H. W. Platta , H. Stenmark , Curr. Opin. Cell Biol. 2011, 23, 393. 21474295 10.1016/j.ceb.2011.03.008

[30] J. Han , K. Pluhackova , R. A. Böckmann , Front. Physiol. 2017, 8, 5. 28163686 10.3389/fphys.2017.00005 PMC5247469

[31] J. Iscaro , H. Yu , N. Martinez , S. Subramaniam , P. Joyce , H. Wang , B. P. Dyett , J. White , C. A. Prestidge , C. J. Drummond , S. Bozinovski , J. Zhai , Adv. Funct. Mater. 2024, 34, 2405286.

[32] J. Li , X. Wang , T. Zhang , C. Wang , Z. Huang , X. Luo , Y. Deng , Asian J. Pharm. Sci. 2015, 10, 81.

[33] T. Kaasgaard , C. J. Drummond , Phys. Chem. Chem. Phys. 2006, 8, 4957. 17091149 10.1039/b609510k

[34] D. P. Siegel , Biophys. J. 2008, 95, 5200. 18805927 10.1529/biophysj.108.140152 PMC2586550

[35] H. Kim , C. Leal , ACS Nano 2015, 9, 10214. 26390340 10.1021/acsnano.5b03902

[36] C. Fong , T. Le , C. J. Drummond , Chem. Soc. Rev. 2012, 41, 12971322. 10.1039/c1cs15148g 21975366

[37] J. B. Strachan , B. P. Dyett , Z. Nasa , C. Valery , C. E. Conn , J. Colloid Interface Sci. 2020, 576, 241. 32428785 10.1016/j.jcis.2020.05.002

[38] Y. Li , L. Gao , X. Tan , F. Li , M. Zhao , S. Peng , Biochim. Biophys. Acta, Biomembr. 2016, 1858, 1801. 10.1016/j.bbamem.2016.04.014 27117641

[39] S. Pollock , R. Antrobus , L. Newton , B. Kampa , J. Rossa , S. Latham , N. B. Nichita , R. A. Dwek , N. Zitzmann , FASEB J. 2010, 24, 1866. 20097877 10.1096/fj.09-145755

[40] F. Szymanowski , A. A. Hugo , P. Alves , P. N. Simões , A. Gómez‐Zavaglia , P. F. Pérez , Colloids Surf., B 2017, 156, 38. 10.1016/j.colsurfb.2017.04.058 28500977

[41] G. Ashby , K. E. Keng , C. C. Hayden , S. Gollapudi , J. R. Houser , S. Jamal , J. C. Stachowiak , ACS Appl. Mater. Interfaces 2023, 15, 49988. 37862704 10.1021/acsami.3c09399 PMC11165932

[42] J. L. Höög , E. Gluenz , S. Vaughan , K. Gull , in Methods in Cell Biology, Vol. 96, Academic Press, San Diego, CA 2010, pp. 175–196. 20869523 10.1016/S0091-679X(10)96008-1

[43] G. Cooper , K. Adams , The Cell: A Molecular Approach, Oxford University Press, Oxford 2022.

[44] G. Guidotti , Arch. Intern. Med. 1972, 129, 194. 5058547

[45] H.‐H. Shen , P. G. Hartley , M. James , A. Nelson , H. Defendi , K. M. McLean , Soft Matter 2011, 7, 8041.

[46] E. Jabłonowska , E. Nazaruk , D. Matyszewska , C. Speziale , R. Mezzenga , E. M. Landau , R. Bilewicz , Langmuir 2016, 32, 9640. 27550742 10.1021/acs.langmuir.6b01746

[47] P. Vandoolaeghe , A. R. Rennie , R. A. Campbell , T. Nylander , Langmuir 2009, 25, 4009. 19714826 10.1021/la802766n

[48] L. Boge , K. L. Browning , R. Nordström , M. Campana , L. S. E. Damgaard , J. Seth Caous , M. Hellsing , L. Ringstad , M. Andersson , ACS Appl. Mater. Interfaces 2019, 11, 21314. 31120236 10.1021/acsami.9b01826

[49] B. D. Chithrani , W. C. W. Chan , Nano Lett. 2007, 7, 1542. 17465586 10.1021/nl070363y

[50] S. C. Boca , M. Potara , F. Toderas , O. Stephan , P. L. Baldeck , S. Astilean , Mater. Sci. Eng., C 2011, 31, 184.

[51] B. P. Dyett , H. Yu , S. Sarkar , J. B. Strachan , C. J. Drummond , C. E. Conn , ACS Appl. Mater. Interfaces 2021, 13, 53530. 34726885 10.1021/acsami.1c09909

[52] A. I. Shevchuk , P. Hobson , M. J. Lab , D. Klenerman , N. Krauzewicz , Y. E. Korchev , Pfluegers Arch. ‐ Eur. J. Physiol. 2008, 456, 227. 18180951 10.1007/s00424-007-0410-4 PMC2270919

[53] M. Benchimol , C. Batista , W. De Souza , J. Submicrosc. Cytol. Pathol. 1990, 22, 39. 2138055

[54] C. R. Quijia , C. C. Bonatto , L. P. Silva , M. A. Andrade , C. S. Azevedo , C. Lasse Silva , M. Vega , J. M. de Santana , I. M. D. Bastos , M. L. B. Carneiro , Materials 2020, 13, 5505. 33276688 10.3390/ma13235505 PMC7730638

[55] W. de Souza , Mod. Res. Edu. Top. Micros. 2007, 77.

[56] C. VataruNakamura , T. Ueda‐Nakamura , W. de Souza , FEMS Microbiol. Lett. 2005, 242, 227. 15621442 10.1016/j.femsle.2004.11.008

[57] J. M. Noble , L. M. Roberts , N. Vidavsky , A. E. Chiou , C. Fischbach , M. J. Paszek , L. A. Estroff , L. F. Kourkoutis , J. Struct. Biol. 2020, 210, 107474. 32032755 10.1016/j.jsb.2020.107474 PMC7067680

[58] J. Hermosilla , A. Alonso‐García , A. Salmerón‐García , J. Cabeza‐Barrera , A. L. Medina‐Castillo , R. Pérez‐Robles , N. Navas , Vaccines 2023, 11, 1635. 38005967 10.3390/vaccines11111635 PMC10675537

[59] O. Vasileva , O. Zaborova , B. Shmykov , R. Ivanov , V. Reshetnikov , Front. Pharmacol. 2024, 15, 1466337. 39508050 10.3389/fphar.2024.1466337 PMC11537937

[60] J. Di , Z. Du , K. Wu , S. Jin , X. Wang , T. Li , Y. Xu , Pharm. Res. 2022, 39, 105. 35080707 10.1007/s11095-022-03166-5 PMC8791091

[61] K. J. Hassett , J. Higgins , A. Woods , B. Levy , Y. Xia , C. J. Hsiao , E. Acosta , Ö. Almarsson , M. J. Moore , L. A. Brito , J. Controlled Release 2021, 335, 237. 10.1016/j.jconrel.2021.05.021 34019945

[62] N. Kimura , M. Maeki , A. Ishida , H. Tani , M. Tokeshi , ACS Appl. Bio Mater. 2021, 4, 1783. 10.1021/acsabm.0c01519 35014524

[63] M. Brust , M. Walker , D. Bethell , D. J. Schiffrin , R. Whyman , J. Chem. Soc., Chem. Commun. 1994, 7, 801.

[64] J. Zhai , S. L. Yap , C. J. Drummond , N. Tran , J. Colloid Interface Sci. 2022, 607, 848. 34536939 10.1016/j.jcis.2021.08.173

