# Supplementary material for: Bypassing Endocytic Barriers: Visualizing Membrane Fusion and Endosomal Escape of Cubic Phase Lipid Nanoparticles (Cubosomes)
Source: Small. 2025 Jul 25;21(44):2502231. doi: 10.1002/smll.202502231 (PMC12590531; doi:10.1002/smll.202502231)
Supplement: Supplementary file 1 — Supporting Information [file SMLL-21-2502231-s001.docx]

# Supplementary Information

# Bypassing Endocytic Barriers: Visualizing Membrane Fusion and Endosomal Escape of Cubic Phase Lipid Nanoparticles (Cubosomes)

Sue Lyn Yap^1^, Chaitali Dekiwadia^1^, Enrico Della Gaspera^1^, Calum J. Drummond*^1^, Charlotte E. Conn*^1^, Nhiem Tran*^1^

^1^RMIT University, 124 La Trobe St, Melbourne VIC 3000, Australia

## Detailed Cubosome Structural and Physicochemical Properties

Cryo-TEM images revealed the internal cubic structure of all cubosomes (Figure S1a). Fast Fourier Transform (FFT) analysis confirmed the Im3m space group with characteristic orientations (Figure S1a, insets). SAXS analysis revealed that R18 cubosomes exhibited a primitive (Im3m) cubic phase, confirmed by characteristic Bragg peaks reflecting the √2, √4, √6 indices of the cubic Im3m space group^2^ (Figure S1b). In contrast, the FRET-based cubosomes showed a mixture of cubic Im3m (√2, √4, √6) and cubic Pn3m (√2, √3, √6, √8 ) cubic phases, while Au_10_NP cubosomes displayed a combination of Im3m (√2, √4, √6) cubic phase and weak hexagonal (√1, √3, √4) reflections^2^ (Figure S1b). The weak hexagonal reflections are likely due to the occasional disruption of the mesophase structure by the incorporated thiol-capped Au_10_NPs. SAXS performed in cell media also confirmed that cubosomes maintained their nanostructure under these conditions (Figure S1b).

The average hydrodynamic diameters of all cubosome formulations ranged between 200 and 250 nm, consistent with the target size range (Figures S1c-d). All cubosome formulations exhibited polydispersity index (PDI) values below 0.24, with the microfluidics formulated R18 cubosomes achieving the lowest PDI of 0.05 (Figure S1d). While microfluidics is generally effective in reducing the PDI of nanoparticles^3^, this was not observed for the FRET-based cubosomes, emphasizing the importance of optimizing formulation protocols for specific systems. Zeta potential measurements indicated that all cubosomes were essentially neutrally charged, with values between -1 mV and -8 mV (Figure S1e).


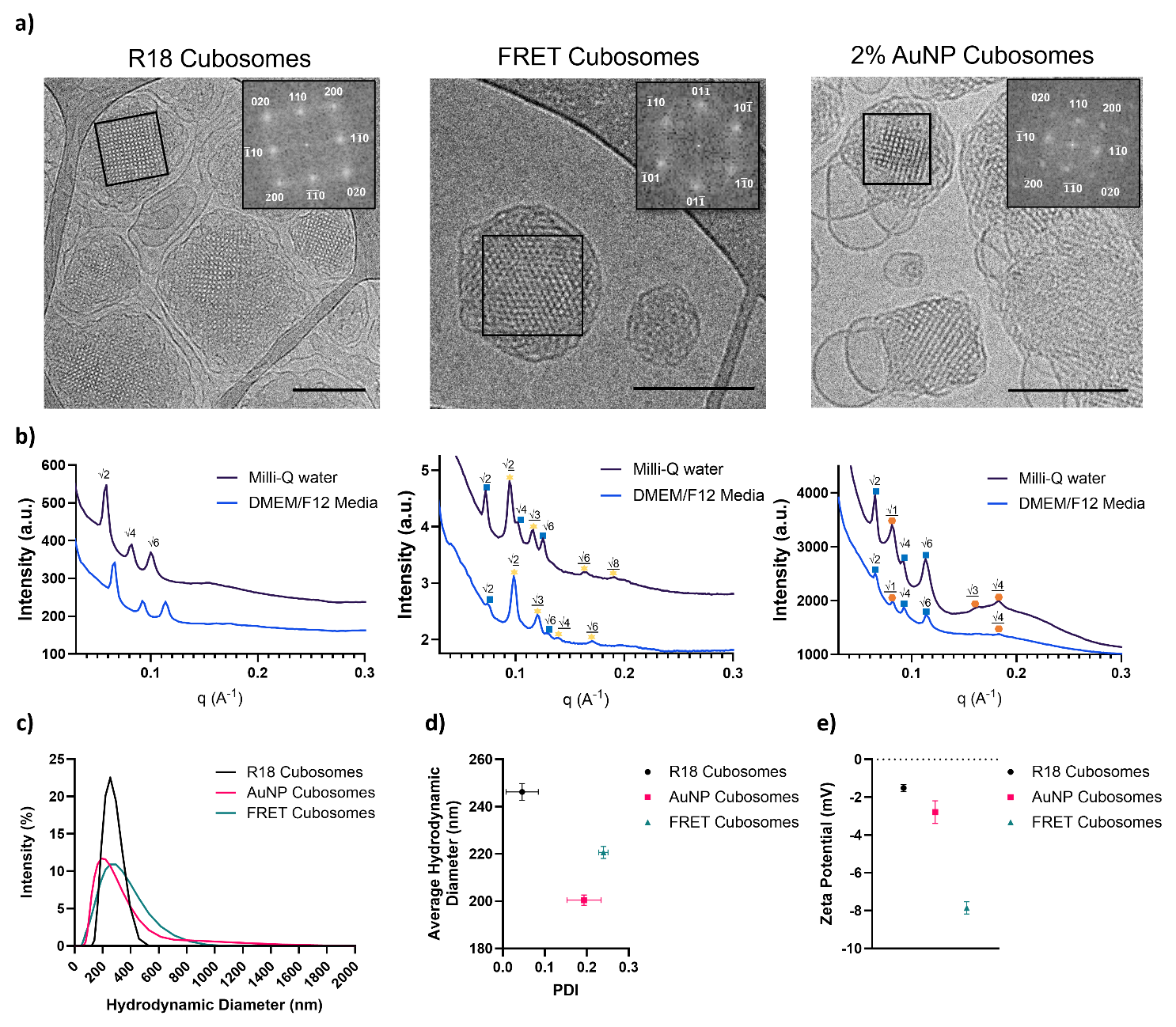


Figure S1 Physicochemical and structural characterization data for all cubosomes used in cell studies. a) Cryo-TEM micrographs of R18 cubosomes, FRET cubosomes, and Au_10_NP cubosomes. FFT analysis was performed on select area (black box) of particles, with insets containing the FFT for the Im3m space groups at different orientations. Scale bar = 200 nm. b) SAXS plots of intensity vs q for R18 cubosomes, FRET cubosomes, and Au_10_NP cubosomes (left to right) in milli-Q water and cell media (incubated for at least 1 hour). R18 cubosomes displayed characteristic Im3m Bragg peaks, FRET cubosomes displayed mixed cubic phases of Im3m and Pn3m, and Au_10_NP cubosomes displayed mixed phases containing Im3m reflections and weak hexagonal reflections. c) Cubosome particle size distributions plots. d) Cubosome average hydrodynamic diameter and polydispersity index (PDI) plots. e) Cubosome zeta potential plots.

## Liposome Formulation and Characterization

**Methods:** R18 labelled liposomes were formulated using the thin film hydration method, as previously detailed in our earlier work^4^. Briefly, stock solutions of DOPC, Pluronic F-127 (10 wt %), and Octadecyl Rhodamine B Chloride (0.1 wt %) were mixed at the appropriate volumes. The lipid mixture was then slowly evaporated using an air outlet to form a thin lipid film around the base of the tube. The thin lipid film was then hydrated with 1mL of Milli-Q water and bath sonicated at 100% amplitude for at least 30 minutes at 60°C. Liposomes were extruded 11 times through a 200 nm polycarbonate membrane for size control. The liposomes were then characterized using DLS, Zetasizer (zeta potential), and cryo-TEM, with the same methods described in the main text.

**Results:** The average hydrodynamic diameter, polydispersity index, and zeta potential for the R18-labeled liposomes were 202.3 nm, 0.171, and -1.08mV, respectively. Cryo-TEM micrographs of R18 liposomes were obtained, demonstrating primarily unilamellar liposomes with the presence of some multilamellar liposomes (Figure S2).


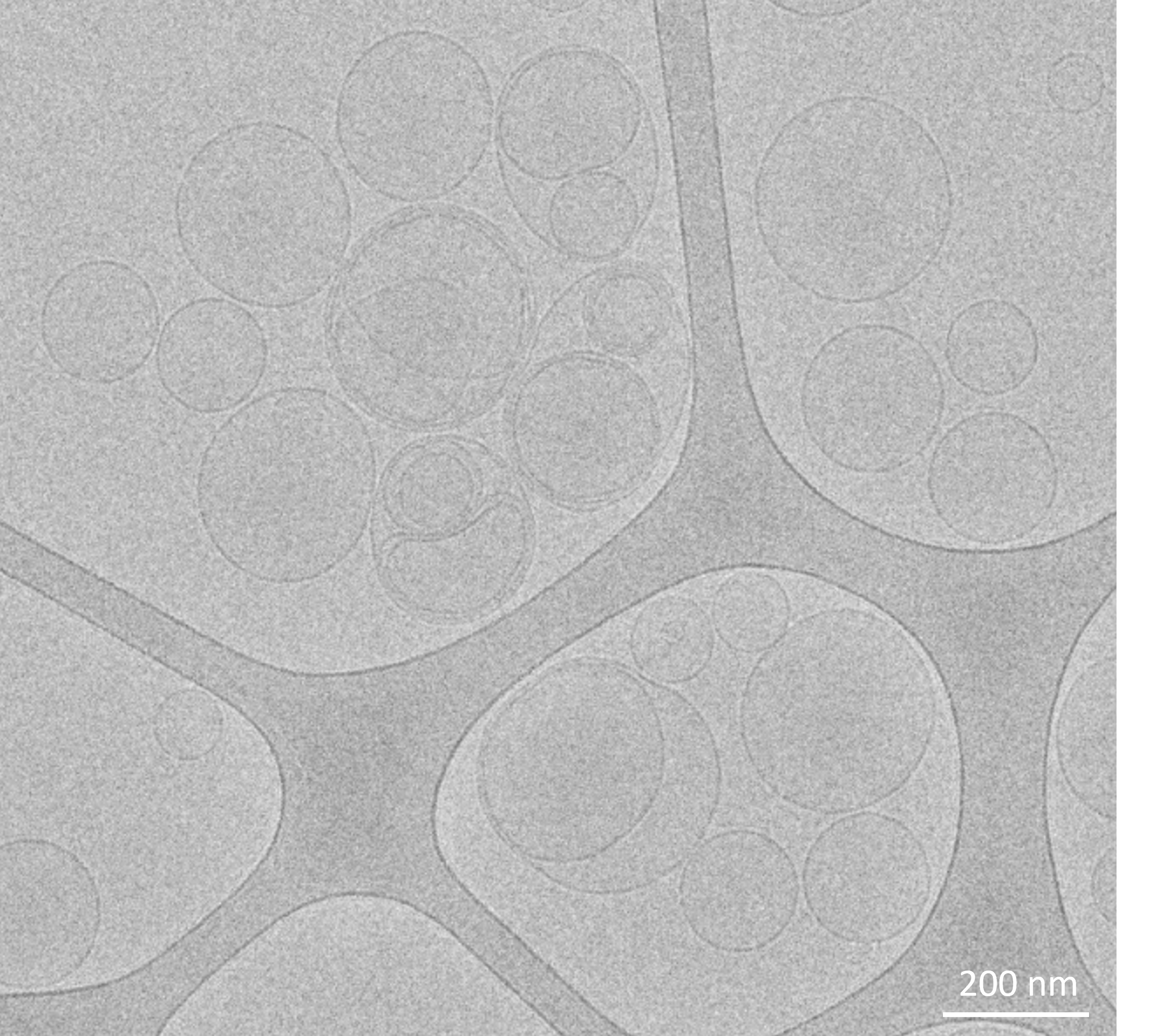


Figure S2 Cryo-TEM micrograph of R18 liposomes.

## Cryo-TEM Micrographs of Mixed-Phase Particles

Cryo-TEM micrographs of mixed phase particles within Au_10_NP and FRET cubosomes were obtained (Figure S3). Disruption of the cubic structure occurs at a high loading of Au_10_NPs in the particles (Figure S3(a)), causing a shift in mesophase from cubic to hexagonal. Both cubic and hexagonal regions can be observed within the same particle. As for FRET cubosomes, a cubosome possessing the Pn3m symmetry is observed (Figure S3 (b)).


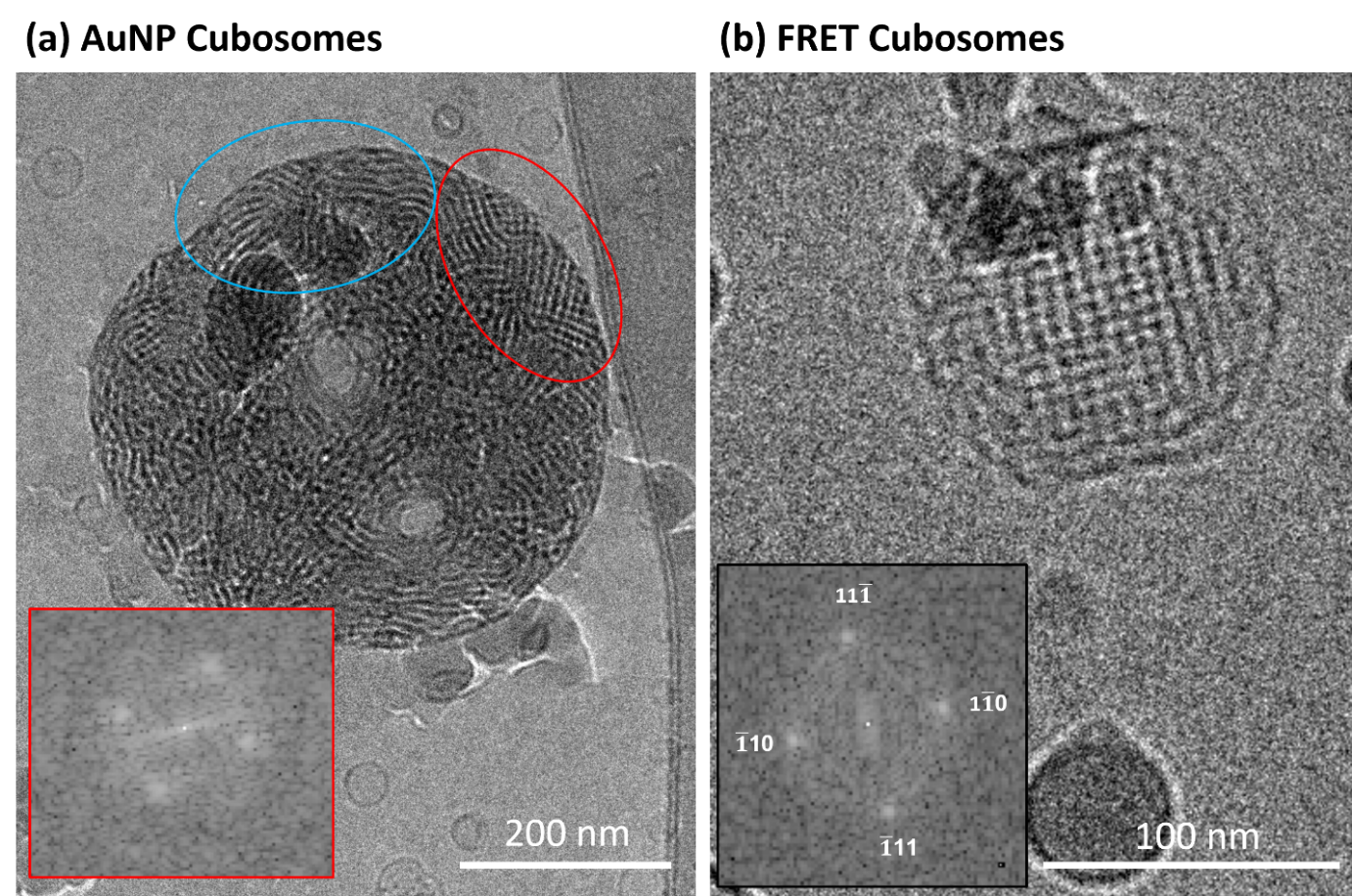


Figure S3 Cryo-TEM images of mixed phase particles within (a) Au_10_NP cubosomes and (b) FRET cubosomes. The region within the red and blue circle represents cubic and hexagonal regions in the Au_10_NP particle respectively. The insets contain the FFT reflections of the cubic regions in the particles.

## Cytotoxicity of Cubosomes

**Methods:** CHO cells were suspended and seeded at 20000 cells/well in a 96-well plate and left to incubate overnight. CHO cells were then washed twice with PBS and various concentrations of cubosomes diluted in DMEM/F12 media in a concentration series were added the cells (between 200µg/mL to 6.25µg/mL) at 200µL per well and left to incubate for at least 2 hours. Untreated cells incubated in DMEM/F12 media were used as a positive control, and media containing 25% DMSO was used as a negative control. After 24 hours, the CellTiter 96® AQueous One Solution Cell Proliferation Assay (MTS assay) and a microplate reader were used to determine cubosome cytotoxicity. 40µL of the MTS assay was added to the cells and left to incubate for 3 hours. The SpectraMax® Paradigm® Multi-Mode Detection Platform microplate reader was used to collect the absorbance data at 490nm and used to calculate the normalized cytotoxicity. All samples were measured in triplicate, and all experiments were repeated in triplicate.

**Results:** The normalized cell viability of FRET-functionalized and 2% Au_10_NP cubosomes were plotted in Figure S4. After 2 h of cubosome treatment, CHO cells were able to tolerate cubosome concentrations of up to 100µg/mL, with cell viability exceeding 79%. For all cell experiments using, treatment times did not exceed 2 hours, therefore a treatment concentration of up to 50µg/mL was deemed acceptable to ensure the health and viability of cells during experiments. Cytotoxicity of rhodamine-labelled liposomes and cubosomes with Chinese Hamster Ovarian (CHO) cells have been previously characterized in our prior work^4^. The acceptable treatment concentration was identified to be 200µg/mL for liposomes, and 25µg/mL for cubosomes, after 24 hours of incubation. The difference in cytotoxicity for liposomes and cubosomes may be attributed to the difference in the cellular internalization and interactions, with cubosomes more likely to interact and fuse with cell membranes causing cytotoxicity by saturation of cubosomes lipids in the cell membrane^5^.


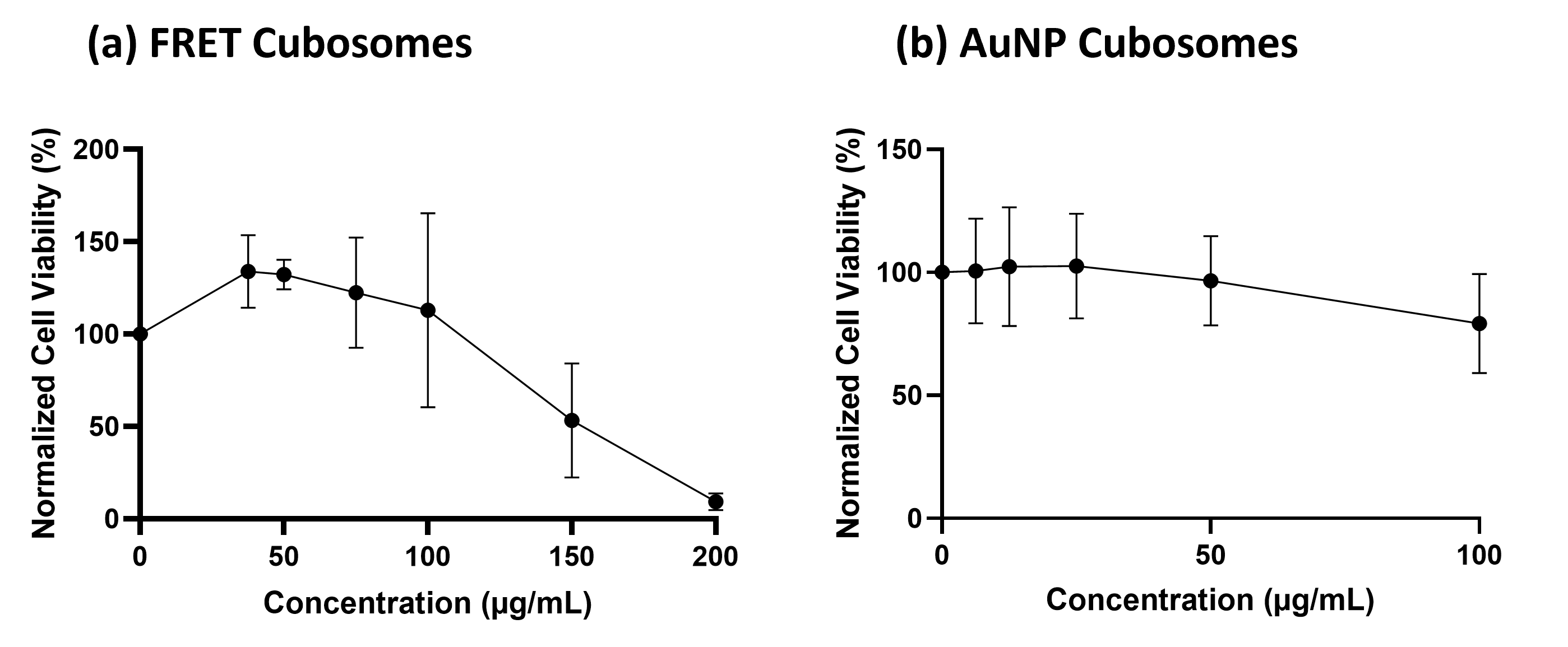


Figure S4 Normalized cell viability a) FRET cubosomes and b) Au_10_NP loaded Cubosomes, after at least 2 hours of incubation N=3. Data = mean ± SD.

## Control Micrographs of FRET Cubosome-Treated Cells

To confirm that the FRET mechanism of cubosomes remains active during treatment and that L-Rhod-PE excitation originates from the FRET process, live cells were imaged 2 hours after FRET cubosome treatment (Figure S5). Cells were first imaged using only the NBD-PE excitation laser (460 nm) and then using both the NBD-PE (460 nm) and L-Rhod-PE (561 nm) excitation lasers. In cells excited solely by the NBD-PE laser, bright red punctate spots, representing intact cubosomes, were visible. In contrast, cells imaged with both lasers showed saturation with both NBD-PE and L-Rhod-PE lipids, with significantly brighter L-Rhod-PE fluorescence directly excited by the L-Rhod-PE laser. Both lasers were set to the same intensity.


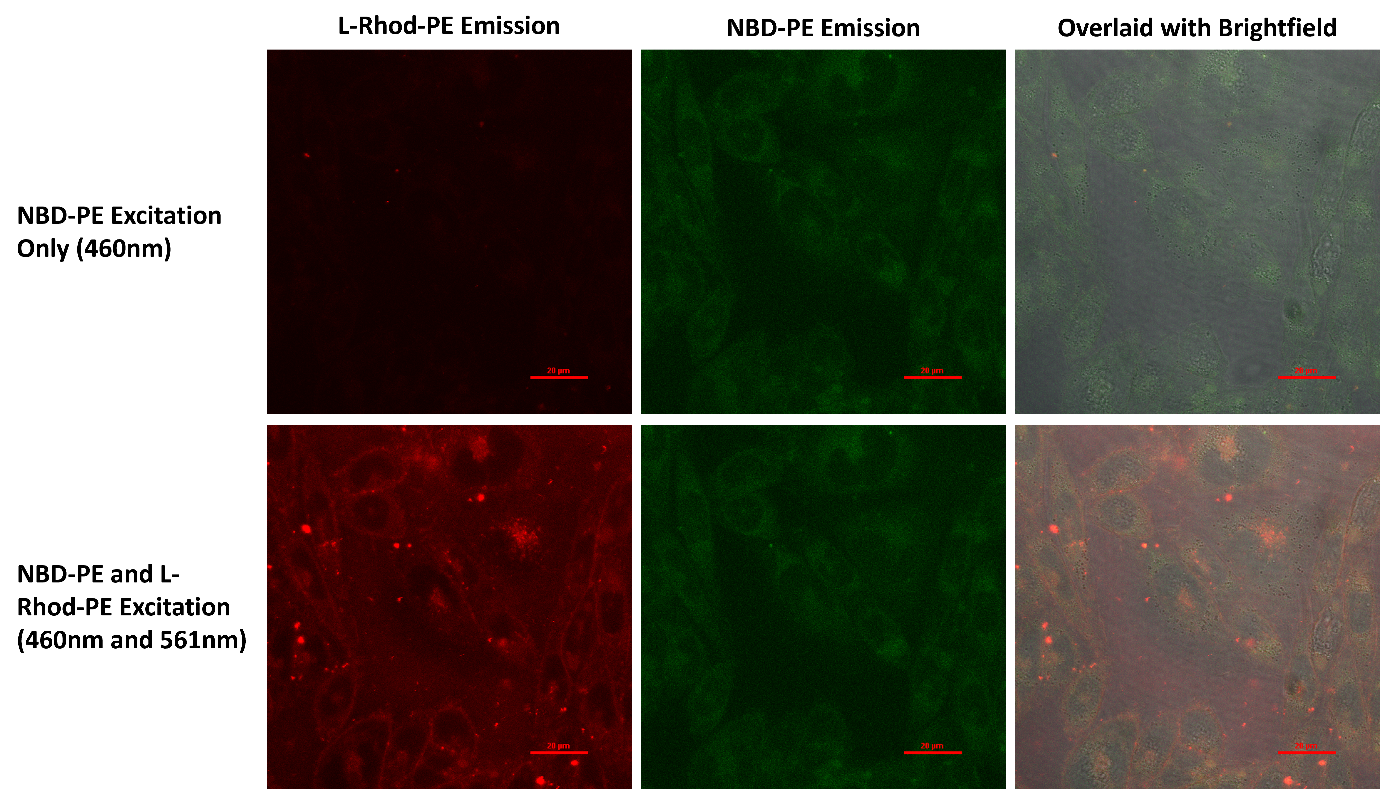


Figure S5 Micrographs of FRET-cubosome-treated cells excited by the NBD-PE laser (460nm) only (above), and micrographs of FRET-cubosome-treated cells excited by both NBD-PE and L-Rhod-PE lasers (460nm and 561nm) (below).

## Additional Micrographs of Single-Particle Tracking of FRET Cubosome-Treated Cells (Endocytic Events)

Additional endocytic events were analysed for their fluorescence intensity changes, demonstrating similar L-Rhod-PE decay (Figure 3) as the cubosomes are processed through endocytosis.


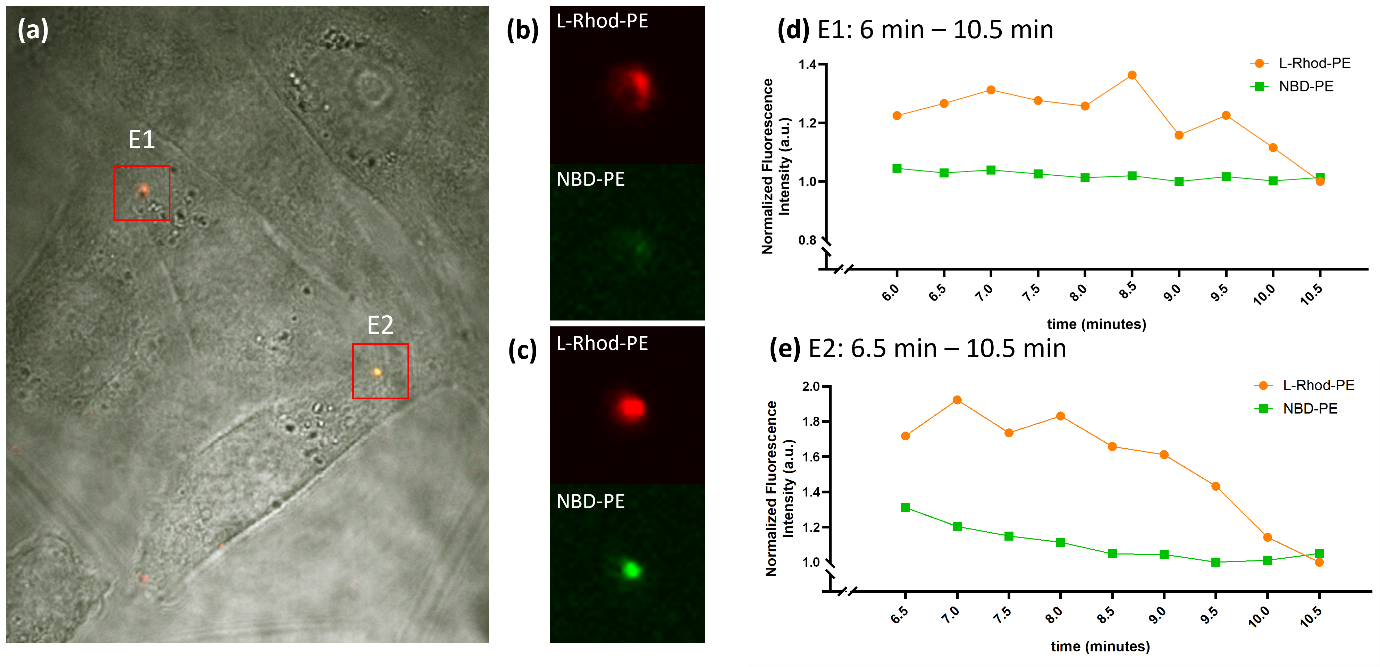


Figure S6 a) The cells where the endocytic events were captured in the cell cytosol. E1 and E2 (red box) represent the endocytic events analysed. b, c) Representative fluorescent images of E1 and E2 with a lifetime of ~4 minutes from t = 6 minutes to t = 20.5 minutes. d,e ) Normalized fluorescence intensity plots of L-Rhod-PE and NBD-PE of the endocytic events E1 an E2 across ~4 minutes.

## Relative Electron Density Micrographs of Au_10_NP Cubosomes

A colour scale was applied to the original cryo-EM micrograph of 10 nm AuNP loaded cubosomes (Au_10_NP) to better illustrate the contrast between the relative electron densities of lipids (yellow-dark green) as compared to the Au_10_NPs encapsulated within the lipid cubosomes (dark blue) (Figure S7).


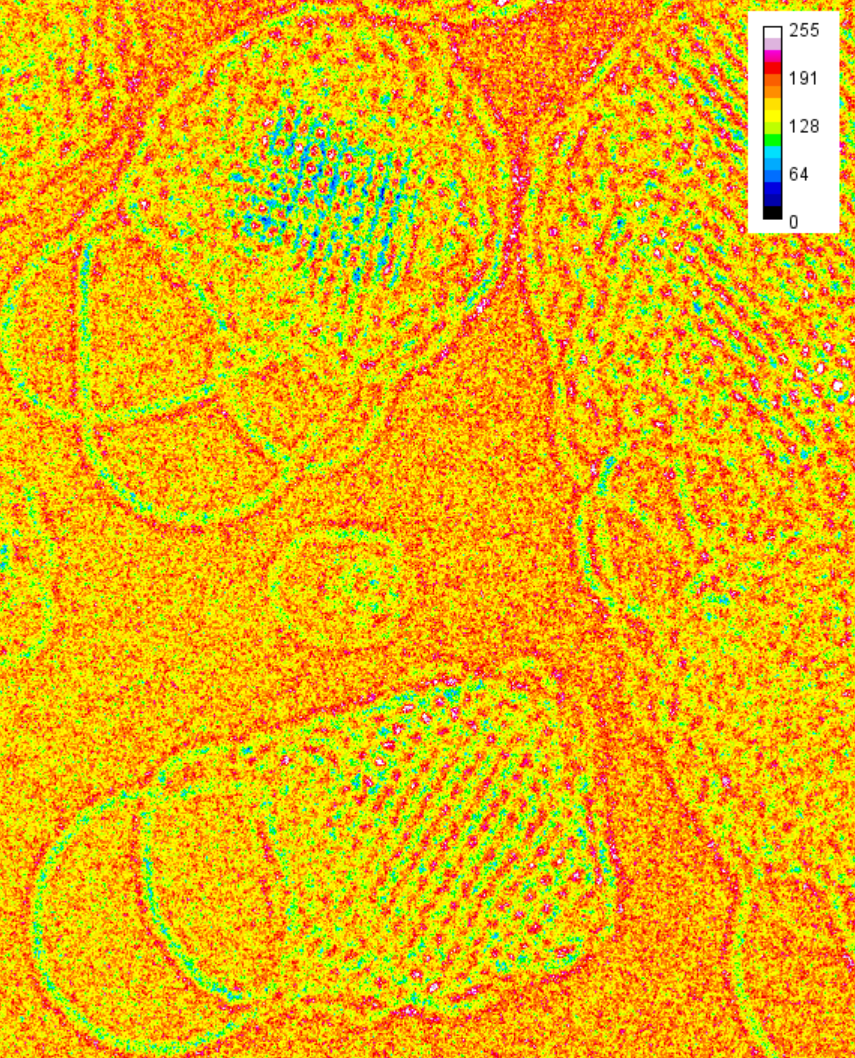


Figure S7 Relative electron density micrographs of 2% Au_10_NP cubosomes.

## Thiol-Capped Gold Nanoparticles (AuNP) Formulation and Characterization

**Methods:** TEM images thiol-capped AuNPs of less than 10 nm were obtained to examine the morphology and size of Au_10_NPs. Briefly, the Au_10_NP hexane formulation was further diluted to a 100µg/mL with hexane. 4µL of the diluted Au_10_NP solution was dripped onto a carbon-coated grid and allowed to dry overnight. The Au_10_NPs were imaged with the Tecnai F30 Transmission Electron Microscope. Au_10_NP diameter and area was analysed using FIJI by analysing a TEM micrograph of Au_10_NPs. Briefly, an image threshold was set to highlight Au_10_NPs and exclude the background, and the ‘Analyse Particles’ function was used to obtain the area and Feret’s diameter of particles. The geometric mean of Feret’s diameter (maximum diameter of a particle) and the minimum diameter to obtain the average diameter of each particle.

**Results:** TEM micrographs of the Au_10_NPs are provided in Figure S8, illustrating the round morphology and small size of Au_10_NPs. The count, average area, average diameter, Feret’s diameter, and minimum diameter are tabulated in Table S1.


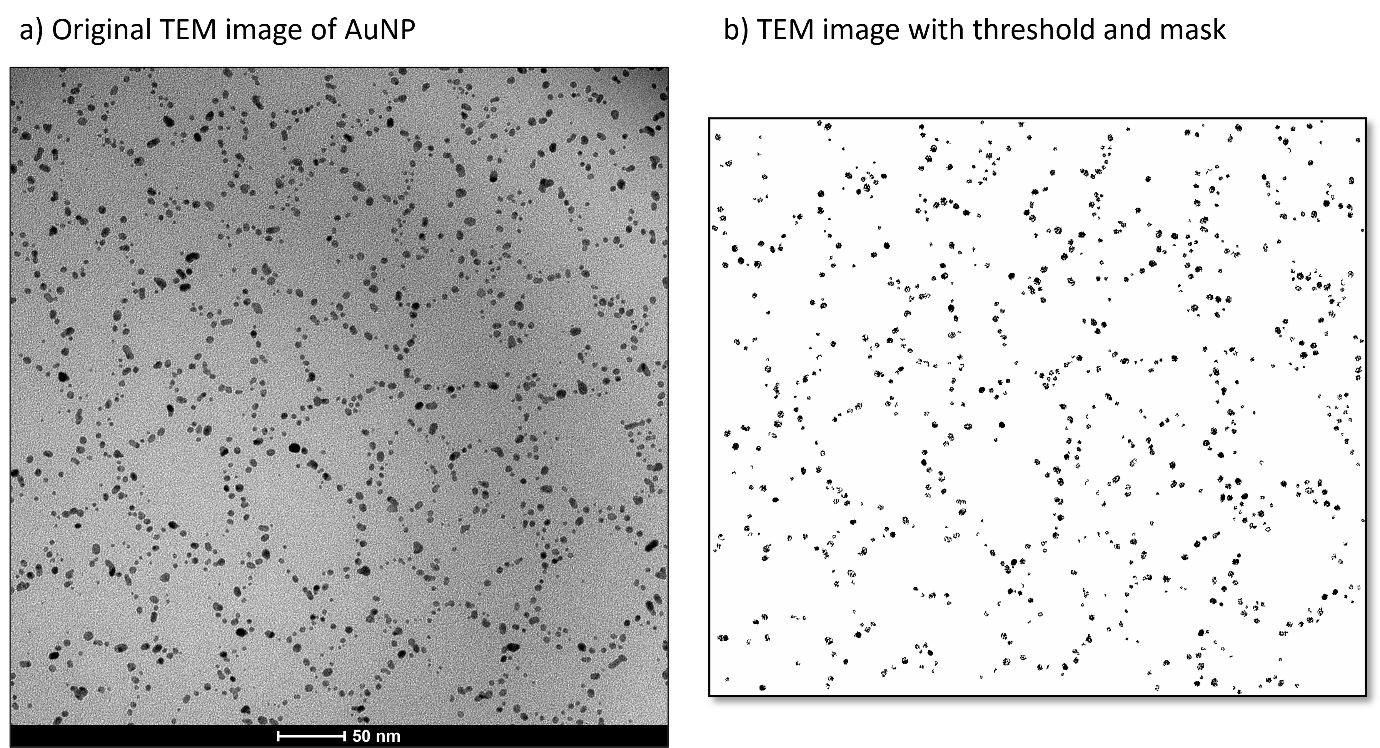


Figure S8 a) TEM Micrograph of Au_10_NPs. b) TEM micrograph after FIJI analysis, with a threshold and mask applied.

Table S1 Characterization of Au_10_NP particle size using image analysis of an Au_10_NP micrograph (Figure S8).

| Count (number of particles analysed) | 757 |
| --- | --- |
| Average area (nm^2^) | 8.35 |
| Average diameter (nm) | 3.86 |
| Feret’s diameter | 4.51 |
| Minimum diameter | 3.33 |

## TEM Micrographs of Untreated Control CHO Cells


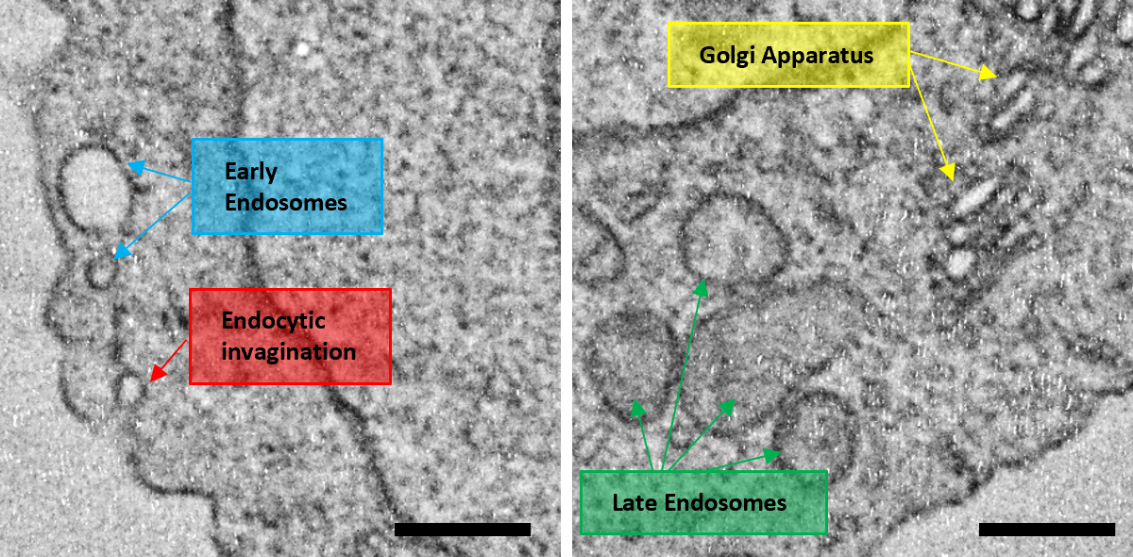


Figure S9 TEM micrographs of untreated control CHO cells. An endocytic invagination and early endosomes (which are predominantly electron lucent^1^) with smooth and round morphologies are seen close to the cell membrane upon initial internalization (left). Late endosomes with smooth and round morphologies, and characterized by a more electron dense appearance^1^, are observed close to the Golgi Apparatus in the cell (right).

## Energy Dispersive X-Ray Spectroscopy (EDS) of Au_10_NP-Cubosome-Treated Cells

EDS was performed during imaging of Au_10_NP-cubosome-treated CHO cells to confirm and contrast the EDS maps of a free Au_10_NP in the cytosol as compared to cellular material (Figure S10). Brightfield Scanning Transmission Electron Microscopy was employed to enhance the contrast of Au_10_NP and cellular structures for EDS mapping (Figure S10(a)). A vesicular structure in the cell is seen next to an Au_10_NP (Figure S10(a)), and EDS mapping was conducted on these two regions (Figures S10 (b) and (c)). The EDS map for the Au_10_NP (spectrum 4) demonstrates the characteristic peaks for gold (Au Mα1 and Au Lα1), which are not present for the vesicular structure/cellular material (spectrum 5).


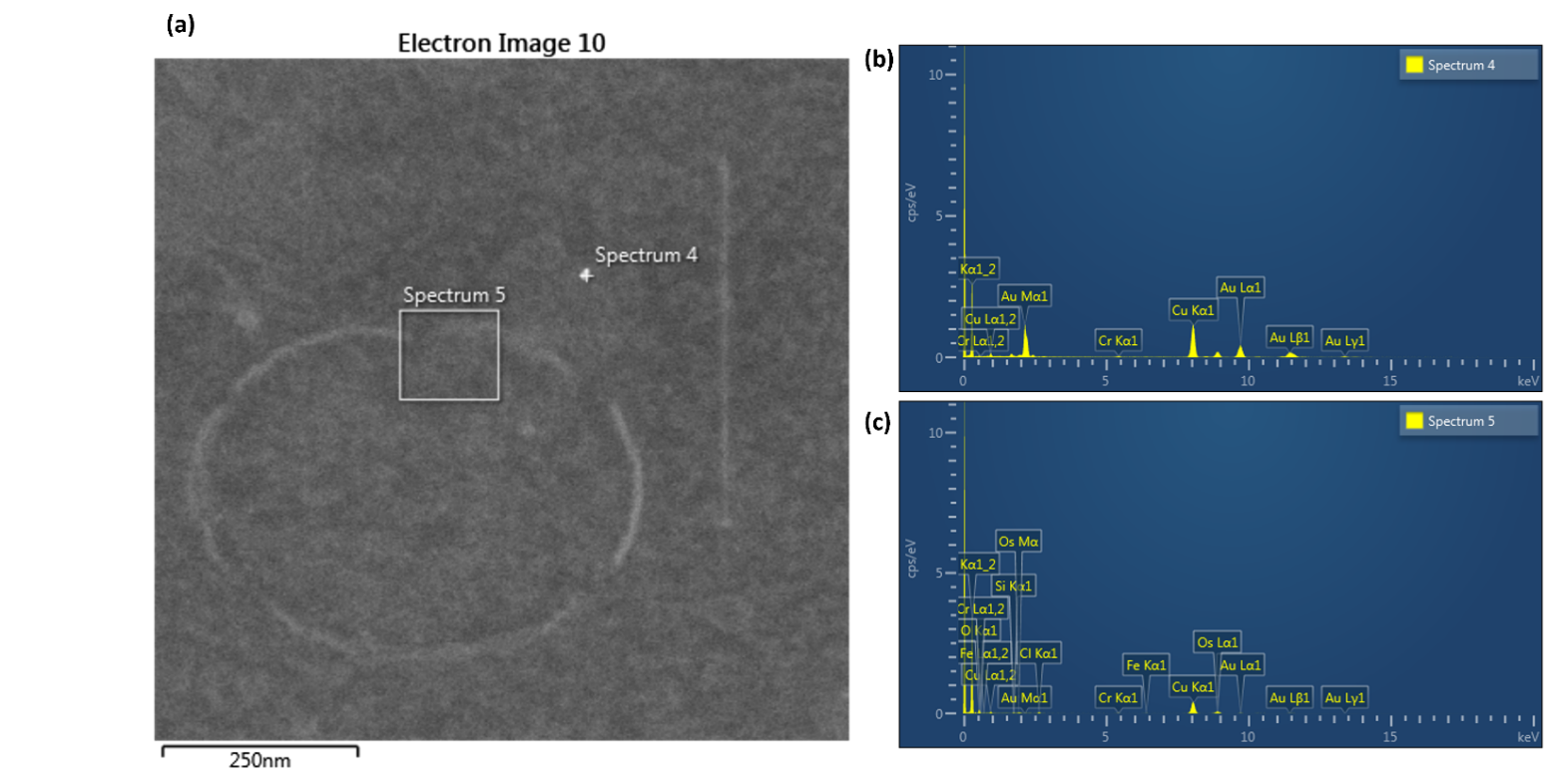


Figure S10 a) Brightfield-STEM micrograph of an Au_10_NP next to a vesicular structure in the cell cytosol of an Au_10_NP cubosome-treated CHO cell. The point and regions selected in spectrums 4 and 5 represent the selected areas for EDS mapping of Au_10_NP and cellular material respectively. b) EDS map of an Au_10_NP showing the characteristic peaks of gold and copper (from the microscope sample holder). b) EDS map of the vesicular structure/cellular material.

## References

1. Klumperman, J.; Raposo, G., The complex ultrastructure of the endolysosomal system. *Cold Spring Harbor perspectives in biology* **2014,** *6* (10), a016857.

2. Kulkarni, C. V.; Wachter, W.; Iglesias-Salto, G.; Engelskirchen, S.; Ahualli, S., Monoolein: a magic lipid? *Physical Chemistry Chemical Physics* **2011,** *13* (8), 3004-3021.

3. Yu, H.; Dyett, B. P.; Zhai, J.; Strachan, J. B.; Drummond, C. J.; Conn, C. E., Formation of particulate lipid lyotropic liquid crystalline nanocarriers using a microfluidic platform. *Journal of Colloid and Interface Science* **2023,** *634*, 279-289.

4. Yap, S. L.; Yu, H.; Li, S.; Drummond, C. J.; Conn, C. E.; Tran, N., Cell interactions with lipid nanoparticles possessing different internal nanostructures: Liposomes, bicontinuous cubosomes, hexosomes, and discontinuous micellar cubosomes. *Journal of Colloid and Interface Science* **2024,** *656*, 409-423.

5. Strachan, J. B.; Dyett, B. P.; Nasa, Z.; Valery, C.; Conn, C. E., Toxicity and cellular uptake of lipid nanoparticles of different structure and composition. *Journal of colloid and interface science* **2020,** *576*, 241-251.
